# Spermine Regulates Pollen Tube Growth by Modulating Ca^2+^-Dependent Actin Organization and Cell Wall Structure

**DOI:** 10.3389/fpls.2017.01701

**Published:** 2017-09-29

**Authors:** Iris Aloisi, Giampiero Cai, Claudia Faleri, Lorella Navazio, Donatella Serafini-Fracassini, Stefano Del Duca

**Affiliations:** ^1^Department of Biological, Geological and Environmental Sciences, University of Bologna, Bologna, Italy; ^2^Department of Life Sciences, University of Siena, Siena, Italy; ^3^Department of Biology, University of Padova, Padova, Italy

**Keywords:** actin dynamics, callose, cell wall, cellulose, cytosolic calcium, pectins, pollen, spermine

## Abstract

Proper growth of the pollen tube depends on an elaborate mechanism that integrates several molecular and cytological sub-processes and ensures a cell shape adapted to the transport of gametes. This growth mechanism is controlled by several molecules among which cytoplasmic and apoplastic polyamines. Spermine (Spm) has been correlated with various physiological processes in pollen, including structuring of the cell wall and modulation of protein (mainly cytoskeletal) assembly. In this work, the effects of Spm on the growth of pear pollen tubes were analyzed. When exogenous Spm (100 μM) was supplied to germinating pollen, it temporarily blocked tube growth, followed by the induction of apical swelling. This reshaping of the pollen tube was maintained also after growth recovery, leading to a 30–40% increase of tube diameter. Apical swelling was also accompanied by a transient increase in cytosolic calcium concentration and alteration of pH values, which were the likely cause for major reorganization of actin filaments and cytoplasmic organelle movement. Morphological alterations of the apical and subapical region also involved changes in the deposition of pectin, cellulose, and callose in the cell wall. Thus, results point to the involvement of Spm in cell wall construction as well as cytoskeleton organization during pear pollen tube growth.

## Introduction

Pollen tube growth represents a remarkable example of polarized expansion, which consists in the deposition of new cell wall material only at the extending tip (Hepler et al., 2013). Newly synthetized ductile methyl-esterified pectins are first secreted at the apex of pollen tubes (O'Neill et al., 1990). After deposition, they are chemically converted into acid pectins at the subapex edge (Rockel et al., 2008) where they bind calcium, thereby contributing to strengthen the cell wall (Palin and Geitmann, 2012; Wolf and Greiner, 2012). This prevents additional deformation of the cell wall and contributes to maintain the cylindrical shape of pollen tubes. In addition to pectins, xyloglucans and arabinogalactan proteins are also secreted, whereas callose and cellulose, the stiff components of the cell wall, are synthetized *in situ* and are absent in the hemispherical apical dome (Cai et al., 2011; Chebli et al., 2012; Mollet et al., 2013). Newly synthetized cell wall components are packed into vesicles and transported along the actin cytoskeleton. They reach the apical domain where they fuse and progressively replace the previously deposited material, which is moved behind the tip by cell expansion (Rojas et al., 2011).

The spatial organization of actin filaments in the pollen tube apex is thus a key factor during pollen tube elongation (Lovy-Wheeler et al., 2005) and its organization is finely modulated by the activity of Rho proteins and actin binding proteins (ABPs) (Qu et al., 2015). ABPs are involved in the correct polymerization and organization of actin filaments by several factors, such as the preference for ADP/ATP-loaded actin, monomeric or filamentous actin, a pH gradient, and a tip-focused Ca^2+^ gradient (Feijo et al., 2001; Hepler et al., 2001; Holdaway-Clarke and Hepler, 2003).

Ca^2+^ gradient is observed at the pollen tube apex and different local Ca^2+^ concentrations along the pollen tube are critical for the cytological organization of growing pollen tubes (Feijo et al., 2001; Steinhorst and Kudla, 2013; Himschoot et al., 2015). Moreover, Ca^2+^ also regulates vesicle fusion and the direction of pollen tube growth (Malho and Trewavas, 1996). An apical influx of Ca^2+^ ions from the extracellular milieu has been firmly established as the main source of this Ca^2+^ gradient. Extensive research has been focused on identifying plasma membrane-located Ca^2+^-permeable channels involved in the observed Ca^2+^ fluxes (Konrad et al., 2011), but also to understand how the ovule regulates Ca^2+^ concentration, thereby regulating pollen tube growth (Ge et al., 2007). Polyamines (PAs) are among the ovular factors that may regulate pollen cytosolic Ca^2+^ levels (Wu et al., 2010; Aloisi et al., 2015). PAs are aliphatic polycations linked to a plethora of physiological processes in plants (Del Duca et al., 2000; Takahashi and Kakehi, 2010; Tiburcio et al., 2014; Cai et al., 2015b). Even if they are found in every plant cell at concentrations ranging from 10 μM to approximately millimolar levels (Bibi et al., 2012), reproductive organs were shown to contain the highest amounts of PAs (Kushad and Orvos, 1990; Alburquerque et al., 2006). The role of PAs in fruit set is not well established, but an influence of these compounds on pollen tube growth and ovule viability has been reported in many *Rosaceae* of agronomical interest. In *Pyrus communis*, it has been shown that PA application at anthesis enhanced pollen tube ovule penetration and delayed ovule senescence (Crisosto et al., 1992). Furthermore, *in vitro* pollen germination and pollen tube growth were stimulated by low or medium PA concentrations in *Prunus mume* (Wolukau et al., 2004). Finally, pollen tube growth in two apricot cultivars was shown to be faster than in other cultivars with less mature ovules having a lower amount of PAs, suggesting an influence of ovule development on pollen tube attraction (Alburquerque et al., 2004).

Pollen also contains high amounts of PAs as well as high activities of their corresponding biosynthetic enzymes (Bagni et al., 1981; Falasca et al., 2010), whose inhibition strongly affects pollen germination (Antognoni and Bagni, 2008). Supplementation of PAs up to sublethal concentrations has been used in the past decades as a tool to understand their role in specific cellular events. We have recently demonstrated that the PA spermine (Spm) can enter through the apex of pear pollen tubes and then spread in the sub-apical region thereby determining enlargement of the apex. These morphological effects induced by exogenous Spm on the *in vitro* growth of pollen tubes are linked to a temporary loss of polarity (Aloisi et al., 2015).

As the effect of exogenous Spm during pollen tube growth seems multifactorial, the purpose of the present work was to study the molecular mechanism by which Spm regulates pollen tube growth, thus fertilization. Emphasis was placed on the role of Spm in regulating Ca^2+^ fluxes and on Ca^2+^-dependent downstream events. As we recently demonstrated that Spm deeply affects pollen tube morphology, the deposition of the main cell wall components (i.e., pectins, callose, and cellulose) was monitored during Spm supplementation. Finally, as cell wall architecture relies on the proper organization of the cytoskeletal apparatus and on vesicular trafficking, these processes were also analyzed.

## Materials and methods

### Chemicals, plant material, and pollen treatment

All chemicals (unless otherwise indicated) were obtained from Sigma–Aldrich (Milan, Italy). Mature pollen of pear (*P. communis* cv. Williams) was collected from plants grown in experimental plots at the University of Bologna (Department of Agricultural Sciences, University of Bologna). Handling, storage, pollen hydration, and germination were performed as previously reported (Bagni et al., 1981; Del Duca et al., 1997).

Pollen viability was tested by MTT (2,5-diphenyl tetrazoliumbromide). The test solution contained 1% concentration of the MTT substrate in 5% sucrose. After 15 min incubation at 30°C the pollen samples were visualized under a light microscope (Nikon Eclipse E600) equipped with a digital camera (Nikon DXM1200). Pollen was considered viable if it turned deep purple.

At 60 min from the start of germination, Spm (100 μM) was added to the growth medium.

The Ca^2+^-channel inhibitors LaCl_3_ and GdCl_3_ were tested at 1, 10, 50, or 100 μM; alternatively, the Ca^2+^-chelating agent EGTA was supplied at 0.4, 1, and 5 mM. These compounds were added to the growth medium 20 min prior to Spm supplementation (i.e., at 40 min of germination).

Pollen tube length was measured using ImageJ software.

For each localization and immunolocalization experiment, at least 30 pollen tubes of approximatively identical length for each developmental stage (control, balloon, snake, and shovel) were imaged and analyzed. For each stage of development, the data shown in figures is highly representative (more than 90% of pollen tubes showed an identical signal distribution).

### Kymograph analysis of pollen tubes

Pollen tubes were observed using a Nikon inverted microscope Diaphot TMD. Extreme care was taken in observing only pollen tubes that grew in line with the focal plane in order to avoid focusing problems. Video clips were captured using a CCD camera C2400-75i Hamamatsu (Hamamatsu Photonics) connected to Argus-20 (Hamamatsu) and converted into MPEG-2 files (25 frames per second) by a video capture system (PCTV Center) working at a resolution of 720 × 576 pixels (Cai et al., 2000). MPEG-2 files were converted into AVI (MJPEG compression) by VirtualDub (http://virtualdub.org/). Video files were opened in ImageJ software (http://rsbweb.nih.gov/ij/index.html) and analyzed by a plug-in kymograph to measure the speed of moving objects in a series of images. The kymograph analyses and measures the gray values in a region of interest (ROI) selected manually for each video frame. A graphical representation of spatial position over time was generated; the X-axis was the time axis (the unit is the frame interval) and the Y-axis indicated the movement rate of each ROI (the unit of measurement is the distance covered by the object expressed in pixels). The speed of objects was measured directly by the plug-in. At least 30 pollen tubes for each sample were analyzed.

### Imaging of pH levels

A BCECF-AM ester probe was used for visualizing proton levels (i.e., pH) in pear pollen tubes (Qu et al., 2012). A final concentration of 5 μM was obtained from a 1 mM dimethyl sulfoxide (DMSO) stock solution; the required volume was directly added to the germination medium containing the re-suspended pollen grains. The cytosolic pH was immediately visualized after addition of the probe to prevent uptake of the pH probe by organelles. In experiments with Spm, the pH probe was either added concomitant with Spm or after progressive 5-min incubation steps following its addition. In doing so, all significant growth stages of pollen tubes after Spm treatment were detected. Samples were observed with a Zeiss AxioImager fluorescence microscope equipped with structured illumination in the FITC filter.

### TAT–aequorin-based Ca^2+^ measurements

Pollen (10 mg) was germinated in growth medium supplemented with 5 μM coelenterazine (Prolume) for 1 h in the dark, washed three times with 3 volumes of fresh medium, and then incubated with 30 μM TAT-aequorin for 10 min in the dark. After extensive washing as above, 100 μl of germinated pollen were transferred in the luminometer chamber. Luminescence measurements were performed with a custom-built luminometer (Electron Tubes Ltd.) containing a 9,893/350A photomultiplier (Thorn EMI), as previously described (Zonin et al., 2011). Ca^2+^ measurement assays were carried out in control conditions (injection, by a light-tight syringe, of an equal volume of germination medium into the sample) or after addition of 100 μM Spm. Luminescence emitted by whole pollen tubes was monitored continuously (every second) for 10 min, and then residual aequorin was discharged by addition of 0.33 M CaCl_2_. The light signal was calibrated off-line into Ca^2+^ concentration values by using a computer algorithm based on the Ca^2+^ response curve of aequorin (Brini et al., 1995).

### Protein extraction, SDS-page, and western blot analysis

To prepare total protein extracts, 1-h germinating pollen samples (10 mg) were lysed with 200 μl of ice-cold Cell Disruption Buffer from the PARIS™ kit (Life Technologies) supplemented with protease inhibitors (0.5 mM phenylmethylsulfonyl fluoride, 1 mM benzamidine, 1 μM leupeptin) in three cycles of 30 s each using a motorized pestle (Sigma-Aldrich), each one followed by 30 s incubation on ice. Samples were then subjected to three cycles of 30 s of sonication (3,510 Branson), each one followed by 30 s incubation on ice. Homogenates were centrifuged at 13,000 *g* for 5 min at 4°C and the protein concentration of the supernatant was determined by the Bradford assay (Bio-Rad). Proteins were separated by SDS-PAGE (12.5% polyacrylamide), transferred onto a polyvinylidene difluoride (PVDF) membrane and processed for immunoblotting, as described by Zonin et al. (2011). A monoclonal anti-polyhistidine antibody (Sigma-Aldrich) was used at a 1:1,500 dilution.

### Actin labeling

For labeling actin filaments, pollen tubes were fixed and permeabilized in 100 mM Pipes buffer, pH 6.9, containing 5 mM MgSO_4_, 0.5 mM CaCl_2_, 0.05% Triton X-100, 1.5% formaldehyde, and 0.05% glutaraldehyde for 30 min according to Lovy-Wheeler et al. (2005). Samples were washed twice with the same buffer, but at pH 7, containing 10 mM EGTA and 6.6 μM Alexa 543-phalloidin (Invitrogen). Samples were placed on slides and covered with a drop of Citifluor as anti-fading agent.

### Monitoring organelle trafficking

Growing pollen tubes were imaged for at least 60 s (or until the organelle was in focus), with frames acquired every 0.5 s. Video clips were captured by the Axiovision software (version 4.3; Zeiss) as ZVI files. Files were then converted into AVI files (MJPEG compression) and then opened in ImageJ software. Image stacks were calibrated with the scale command using the scale bar generated by the Axiovision software. Image (stack) sequences were analyzed by ImageJ using the Manual Tracking plug-in and the trajectories of selected organelles were superimposed onto the initial frame. For each organelle, the absolute velocities were calculated and averaged over the entire trajectory.

### Cell wall staining for pectin, callose, and cellulose

Labeling of cell wall-secreted material was performed using propidium iodide (PI). Evidence that PI labels pectins has been previously described (Rounds et al., 2014; Parrotta et al., 2015). As reported, PI fluorescence matches the fluorescent signal of GFP-labeled pectin methyl esterase; PI also competes with Ca^2+^ in binding to pectins, suggesting that PI binds pectin and can be used as a dye for pectins. Callose and cellulose were labeled as previously described (Cai et al., 2011) using Aniline Blue and Calcofluor White, respectively. Measurement of PI, Calcofluor White, and Aniline Blue fluorescence was performed along the cell edge using the Segmented Line tool of ImageJ. The selection width was about 2 μm (adequate to cover the fluorescence signal). Several pollen tubes of almost identical length were measured. The background was measured outside pollen tubes and subtracted from the average of measurements. As a control of pectin labeling with PI, we also monitored the distribution of high and low methyl-esterified pectins by standard antibodies such as JIM7 and JIM5. The method used was previously described (Del Duca et al., 2013). JIM5 and JIM7 antibodies (obtained from PlantProbes, http://www.plantprobes.net) were used at dilution of 1:50. As a secondary antibody, an Alexa 595 conjugated goat anti-rat IgG (Invitrogen) was used at dilution of 1:50.

### Immunocytochemical analysis of callose synthase

Indirect immunofluorescence microscopy was performed according to standard procedures (Cai et al., 2011). Briefly, samples were fixed with 3% paraformaldehyde in PM buffer (50 mM PIPES, pH 6.9, 1 mM EGTA, 0.5 mM MgCl_2_) for 30 min, washed with PM for 10 min and then incubated with 1.5% cellulysin (Sigma) for 7 min in the dark. After two washes in PM buffer, samples were incubated with the primary antibodies to callose synthase (Cai et al., 2011) at a dilution of 1:50. Antibodies were incubated at 4°C overnight. After two washes with PM buffer, samples were incubated in the dark with a goat anti-rabbit secondary antibody conjugated to Alexa Fluor 488 (Invitrogen) diluted 1:150 for 45 min. After two washes in PM buffer, samples were placed on slides and covered with a drop of Citifluor. Observations were made using a microscope Zeiss AxioImager equipped with structured illumination and a 63x objective; images were captured with an AxioCam MRm camera using the software AxioVision. In controls, primary antibodies were omitted.

### Treatment with microtubule inhibitors and depolymerizing agents taxol, oryzalin, and brefeldin A

Oryzalin at 1 μM was used in combination with Spm and treated samples were observed after 30–60 min; the concentration used is able to depolymerize most microtubules in pollen tubes (Åström et al., 1995; Gossot and Geitmann, 2007). Taxol at 10 μM, a concentration known to stabilize microtubules in pollen tubes (Åström, 1992), was supplied in combination with Spm for 30–60 min. Brefeldin A (BFA) was used at 5 μg/ml^−1^ as previously described (Rutten and Knuiman, 1993; Parton et al., 2003) and observations were made after 30–60 min. All drugs were prepared as concentrated stock solutions in DMSO. In controls, pollen tubes were analyzed either in standard medium or in medium supplemented with equivalent concentrations of DMSO. No differences were observed.

**Figure 1:**
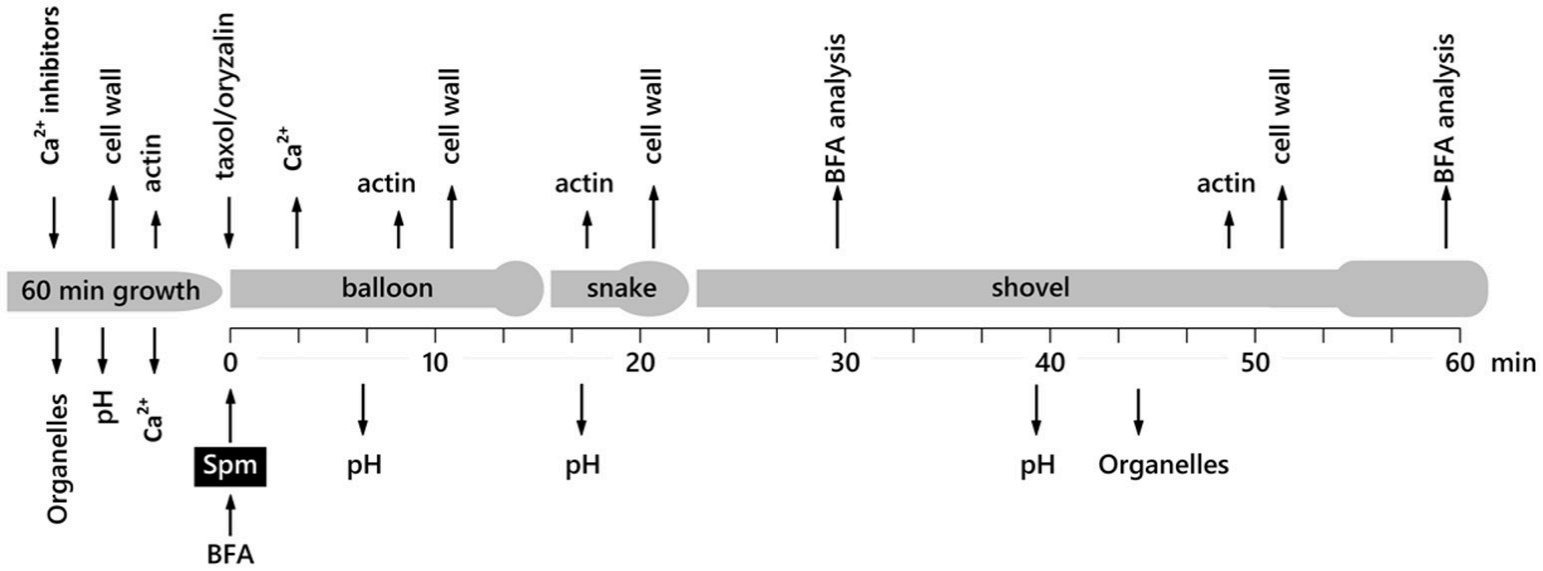
Time scale of the various morphological stages of pear pollen tubes after treatment with Spm and of the main analyses performed.

An overview of the experimental procedure is illustrated in Figure 1.

### Statistical analysis

Differences between data sets were determined by two-way analysis of variance (ANOVA), with a threshold *P*-value of 0.05, performed using GraphPad Prism.

## Results

### The growth speed and shape of Spm-treated pollen tubes are altered

**Figure 2:**
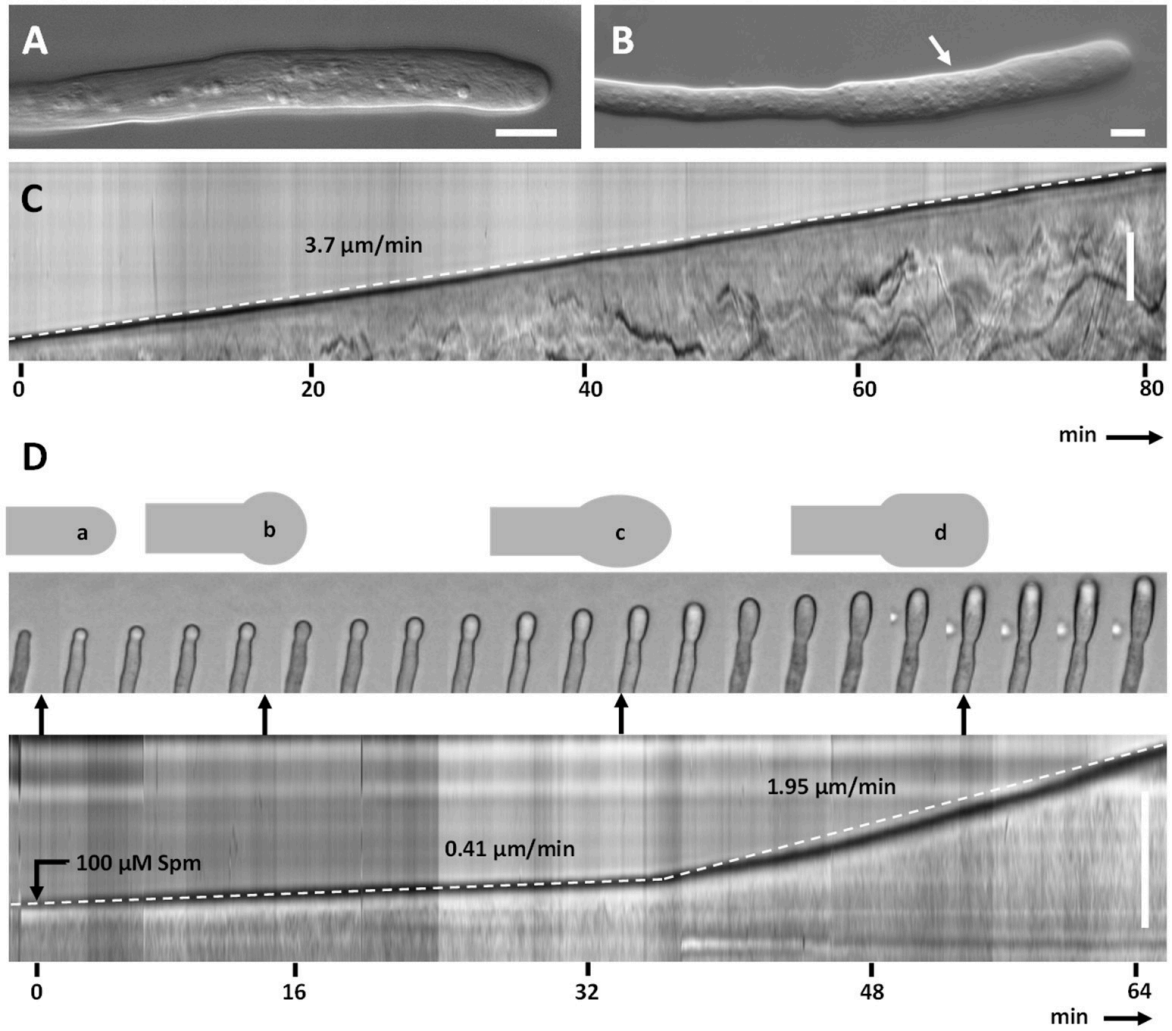
Kymograph analysis of pear pollen tubes after Spm treatment. **(A)** DIC view of a control pollen tube. **(B)** A typical enlarged pollen tube after Spm treatment. Bars: 20 μm. **(C)** Control pollen tube analyzed by kymograph. The dotted line indicates the linearity of growth. The X-axis is the time in minutes, while the Y-axis is the distance covered by the tube tip (scale bar for Y-axis is 100 μm). **(D)** (top part) Video frames showing the effects induced by Spm on a pollen tube. Cartoons indicate the main morphological stages that characterize pollen tubes after Spm treatment; **(a)** no Spm; **(b)** balloon shape; **(c)** snake shape; **(d)** shovel shape (bottom part). Kymograph analysis of a Spm-treated pollen tube. Spm was added at time zero. Scale bar for Y-axis is 500 μm. Small black arrows connecting the kymograph with video frames indicate approximately the time characterized by the four pollen tube shapes. Data are representative of six independent experiments.

Untreated pear pollen developed a regular cylindrical tube (Figure 2A). The diameter of control pollen tubes was 7.5 (±0.7) μm while it increased up to 10.6 (±0.6) μm in the enlarged portions of treated pollen tubes (*n* = 50; Figure 2B, arrow). The kymograph revealed a constant growth velocity of 3.7 (±1.1) μm min^−1^ in control pollen tubes (Figure 2C). When treated with Spm, pollen tubes were characterized by a lower growth velocity, 0.41 (±0.15) μm min^−1^; moreover, the tube apex isotropically enlarged into the so-called balloon stage within the first 15 min (Figure 2D). The apex then modified its shape and entered the so-called “snake stage” and after 20–30 min it developed into an enlarged tubular form, described as “shovel stage” (Figure 2D). Concomitantly, the growth speed increased to 1.95 (±0.7) μm min^−1^, but never recovered the original growth rate. For each measurement of growth speed, at least 50 pollen tubes were analyzed. In all cases, the differences in diameter of pollen tubes before and after spermine treatment were statistically significant, as well as differences in growth rates before and after treatment (*p* < 0.01, student test).

### Spm treatment alters both cytosolic Ca^2+^ concentration and the pH gradient

**Figure 3:**
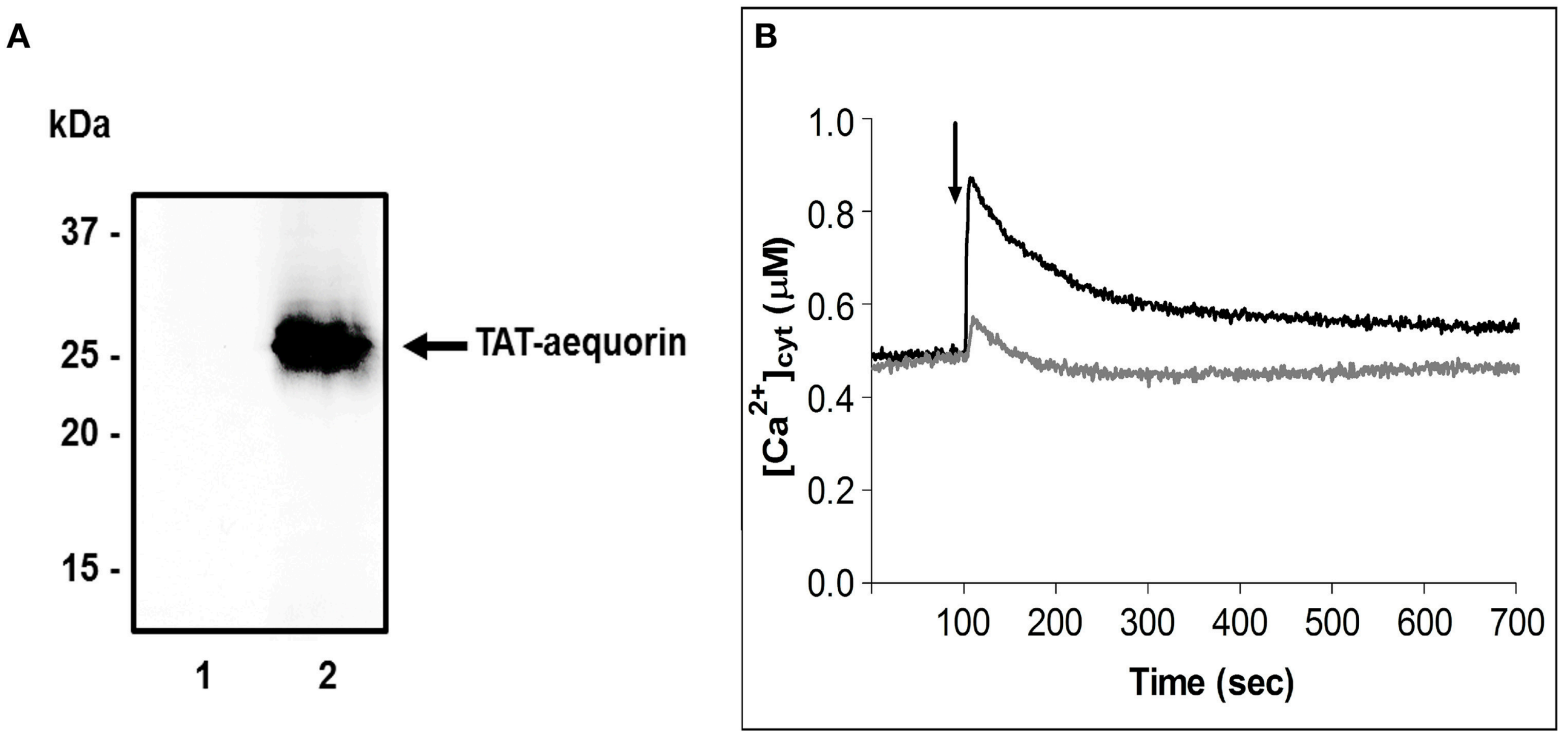
Spm affects cytosolic Ca^2+^ concentration in germinating pear pollen. **(A)** Immunoblot analysis assessing the delivery of TAT-fused aequorin into germinating pollen. Samples were incubated in the absence (1) or presence (2) of TAT-aequorin (30 μM) for 10 min. Total protein extracts (30 μg) were separated by 12.5% SDS-PAGE, transferred onto PVDF and incubated with an anti-polyHis antibody. **(B)** Monitoring of cytosolic Ca^2+^ dynamics in germinating pollen by using TAT-aequorin. Cytosolic free Ca^2+^ concentration ([Ca^2+^]_cyt_) was measured after 10 min incubation of germinating pollen with TAT-aequorin and subsequent challenge (arrow, 100 s) with either the germination medium (gray trace) or 100 μM Spm (black trace). Ca^2+^ traces are representative of three independent experiments that gave very similar results.

To analyze and precisely quantify the alteration in Ca^2+^ fluxes induced by Spm in germinating pollen, Ca^2+^ dynamics were monitored in pear pollen tubes by using the TAT-aequorin method (Zonin et al., 2011). The translocating properties of the cell-penetrating peptide TAT were used to deliver into germinating pollen tubes the covalently-linked bioluminescent Ca^2+^ reporter aequorin. Western blot analyses of total protein extracts from 1 h germinated pollen, incubated for 10 min with the TAT-aequorin fusion protein (30 μM), confirmed the internalization of the recombinant protein, which was absent in control samples (Figure 3A). TAT-aequorin-based Ca^2+^ measurement assays demonstrated that the cytosolic free Ca^2+^ concentration ([Ca^2+^]_cyt_) in germinating pear pollen was maintained at about 0.50 μM (Figure 3B). Upon treatment with 100 μM Spm, pear pollen tubes responded with a rapid [Ca^2+^]_cyt_ increase, that reached a peak value of about 0.90 μM after 20 s and then gradually decreased to almost basal values within 10 min (Figure 3B, black trace). On the other hand, the mechanical perturbation caused by the injection of an equal volume of germination medium (touch control) induced only a modest [Ca^2+^]_cyt_ change characterized by a very limited amplitude (about 0.55 μM, Figure 3B, gray trace). Notably, the recorded Ca^2+^ levels represent mean values between [Ca^2+^]_cyt_ at the tip and at the base of pollen tubes, because the aequorin method allows monitoring Ca^2+^ dynamics along the entire length of the pollen tube. After 2 h of pollen germination, a two-fold increase in the basal [Ca^2+^]_cyt_ was observed, with a concomitant loss of responsiveness to Spm, in agreement with a physiological and progressive decrease of pollen viability (Supplementary Figure 1).

**Figure 4:**
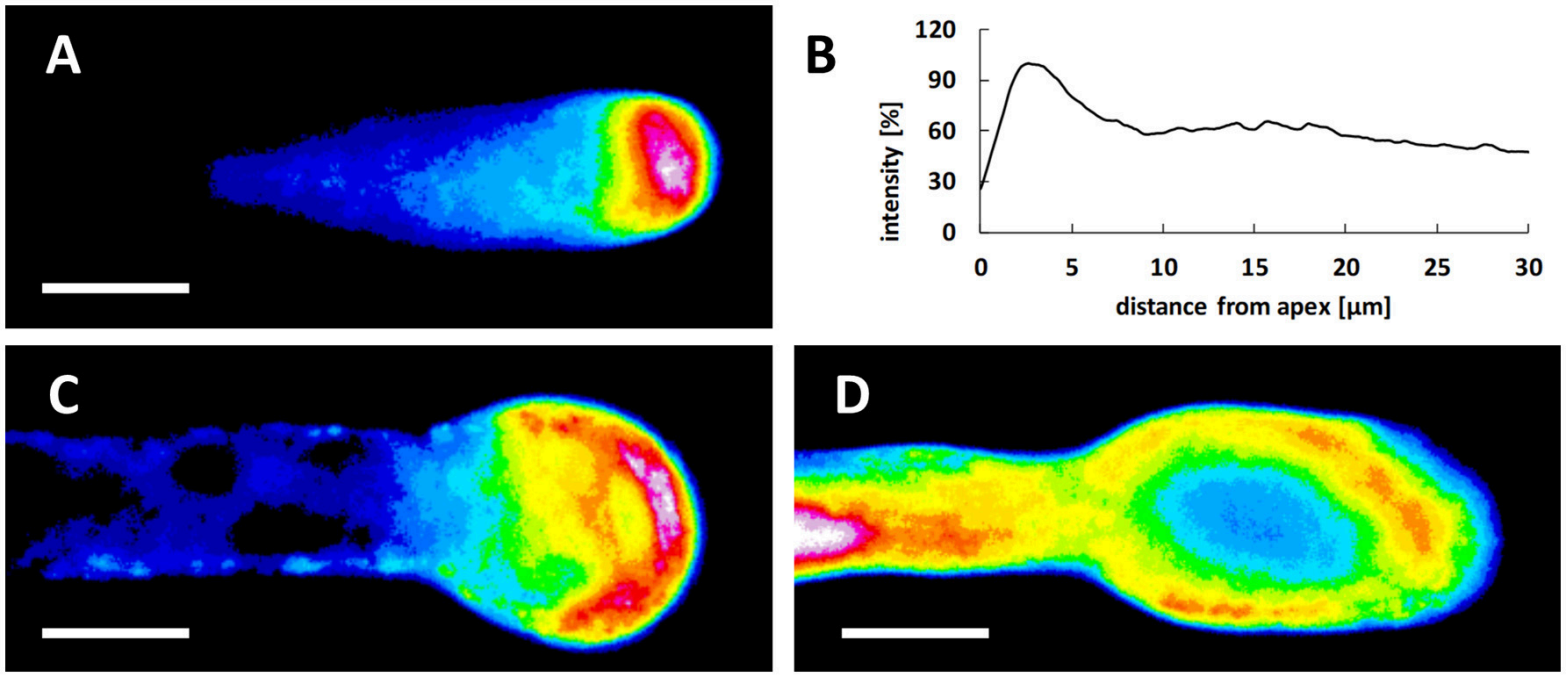
Treatment with Spm dramatically modifies the pH gradient at the pear pollen tube apex. **(A)** In controls, pollen tubes have a tip-oriented pH gradient with an acidic region located at the tube apex. The pH acid region disappears shortly after 5–10 μm from the apex and only a weak background signal can be observed. **(B)** The data is confirmed by the relative measurement of the fluorescence intensity that is representative of three independent experiments. **(C)** At the balloon stage of Spm-treated pollen tubes, the pH gradient changes and the highest H^+^ concentration is redistributed throughout the surface of the swelling apex. **(D)** At the shovel stage, H^+^ concentration is approximately homogeneous throughout the new enlarged pollen tube. No H^+^ accumulation was observed at the apex of the new pollen tube. Bar = 10 μm.

To check the effect of Spm while avoiding the influx of external Ca^2+^, lanthanum (La^3+^) and gadolinium (Gd^3+^) were supplied 20 min before the PA. When these compounds were added to the germination medium (containing 1.27 mM Ca^2+^), no morphological changes were recorded over 2 h, including no tip swelling or increase in tube diameter (Supplementary Figure 2). By contrast, tube elongation was significantly inhibited by addition of 10, 50, or 100 μM La^3+^ or Gd^3+^ and 0.4, 1, 5 mM EGTA after the first hour of germination (Supplementary Table 1). Moreover, pretreatment with La^3+^, Gd^3+^, and EGTA counteracted, in a dose-dependent manner, the extension of the shovel-shaped apical region and thus the effects of Spm (Supplementary Figure 1). In addition to the Ca^2+^ gradient, the proton gradient is also a distinctive feature of growing pollen tubes and a low pH value at the tube tip is supposed to be necessary for optimal growth. Pollen tubes of some plant species (e.g., *Lilium longiflorum*) are also characterized by an increase in pH behind the tip domain (the so-called alkaline band; Feijo et al., 1999). However, tobacco does not display a distinct alkaline band (Michard et al., 2008, 2017). In growing pear pollen tubes, the clear zone was demonstrated to be acidic and the subapical zone alkaline (Qu et al., 2012). We therefore analyzed potential changes in pH values during Spm treatment at different times. In control pollen tubes, the lowest pH values were observed in the extreme tube region (the tip). The peak of fluorescence, representing the zone with the lowest pH, covered approximately the first 5 μm of the tip region; the signal then dropped rapidly and stabilized at background values (Figures 4A,B). Spm-treated pollen tubes at the balloon stage displayed the highest proton concentration in a wider area at the apex, in accord with the new cell shape (Figure 4C). During transition to the so-called “shovel shape,” i.e., when pollen tubes resumed growth, the proton gradient was dissipated. Indeed, areas with very low pH could hardly be observed at the apex. Instead, a relatively high concentration of protons was visible throughout the pollen tube whereas low pH areas were observed in distal regions (Figure 4D).

### Spm induces changes in the actin cytoskeleton of growing pollen tubes

**Figure 5:**
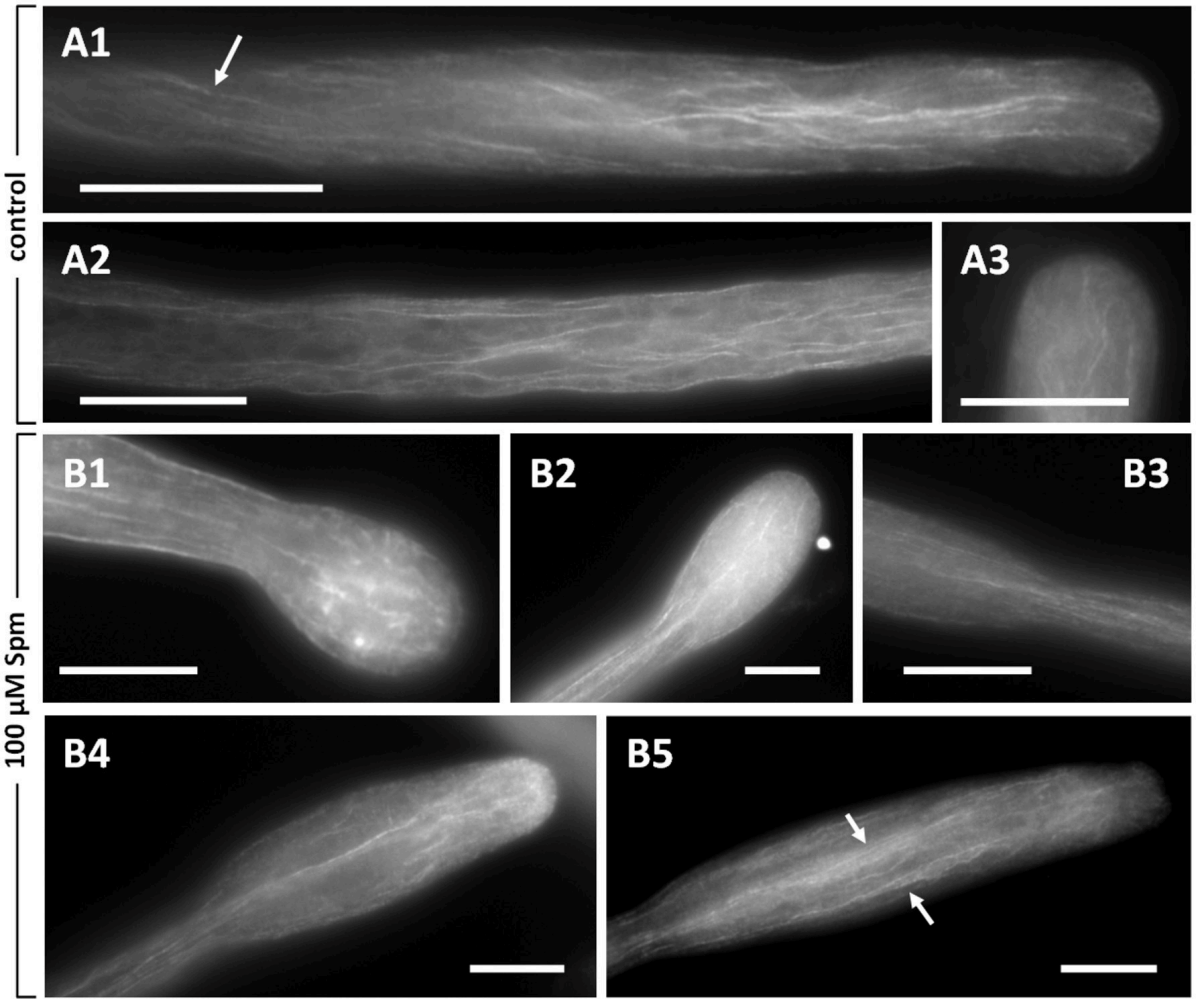
Distribution of actin filaments in pear pollen tubes. **(A1,A2)** Control pollen tubes. The arrow indicates a longitudinal helicoidally-arranged actin filament. **(A3)** Detail of a control pollen tube apex. **(B1–B5)** Pollen tubes treated with Spm. **(B1)** A balloon-shaped pollen tube showing completely disorganized actin filaments in the apex. **(B2)** Reorganization of actin filaments at the snake-shaped stage. **(B3)** When the pollen tube re-starts to grow, actin filaments run along the cortical region and open like a fan at the neck level. **(B4,B5)** Actin filaments can be observed as the new shovel-shaped pollen tube resumes growth. Arrows indicate some actin bundles that run longitudinally along the middle and the cortex of pollen tubes. Bars = 10 μm. Data are representative of three independent experiments.

Perturbation of pollen tube growth might suggest that changes to the cytoskeleton also occurred. Moreover, because the Ca^2+^ gradient is likely involved in the formation of the swollen pollen tube tip and a key regulator of cytoskeleton dynamics, the first attempt was to analyze the actin cytoskeleton. Figures 5A1,A2 illustrates typical images of actin in pear pollen tubes visualized by fluorescence microscopy. A delicate pattern of longitudinal actin filaments is present in the shank of pollen tubes. Actin filaments occasionally showed a helical arrangement (arrow in Figure 5A1) and extended up to the tube tip; nevertheless, the very tip region was always devoid of prominent actin labeling. In addition, actin in the tip appeared to be less organized and neither fringe-like nor collar-like structures were discernible (Figure 5A3).

When pollen tubes were treated with Spm, changes to the actin cytoskeleton mirrored the changes in morphology and growth rate. Thus, the subapical actin network lost its level of organization and filaments appeared shorter and disorganized compared to controls; furthermore, the presence of several fluorescent spots suggested aggregation and depolymerization of actin (Figure 5B1). In the subapical region, actin filaments appeared as partially damaged while in the shanks they seemed relatively normal. Therefore, when Spm-treated pollen tubes assumed the balloon shape, the actin array in the apical dome appeared as disordered, which likely correlates with the dissipation of the Ca^2+^ gradient and the isotropic expansion of the tubes. An ordered pattern of actin filaments was partially restored as soon as pollen tubes resumed growth and the pollen tube apex changed from the balloon into the snake shape. In this new condition, a delicate fibrillar pattern might form in the expanded tip, with actin fibrils penetrating the new apex (Figure 5B2). Moreover, actin filaments in the new developing tube underwent conformational changes consisting of actin filaments coming from the distal tubular portion (not enlarged) that penetrated in the expanded portion of pollen tubes and dispersed thereby adapting to the new shape (Figure 5B3). This new pattern of actin filaments was more evident when pollen tubes started assuming a shovel shape (Figure 5B4) and became progressively clearer while the shovel-shaped pollen tube expanded (Figure 5B5). In the expanded portion of the tube, actin filaments formed bundles that were seemingly thicker in central and cortical regions (arrows in Figure 5B5). Actin filament bundles in the unchanged part of the pollen tube shank did not seem to be affected by Spm treatment.

### Microtubules are not apparently involved in establishing the new growth pattern

**Figure 6:**
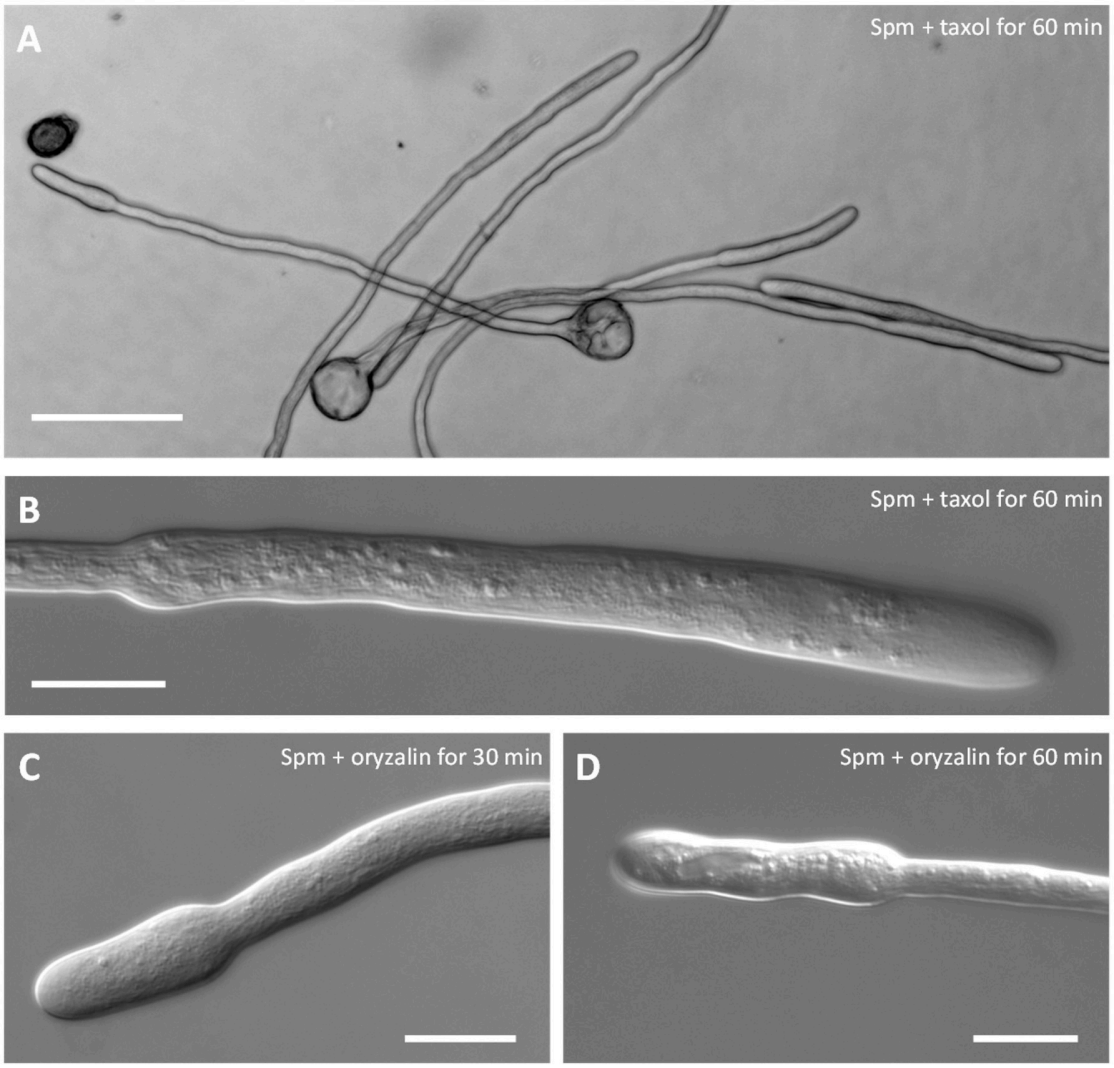
Co-treatment of pear pollen tubes with Spm and microtubule inhibitors. **(A)** DIC view of pollen tubes after co-treatment with Spm and taxol. **(B)** Detail of a single pollen tube after Spm+taxol treatment (DIC view). **(C,D)** DIC views of pollen tubes after co-treatment with Spm and oryzalin. Neither inhibitor affected the formation of the shovel shape. Images were captured after the incubation time indicated in each picture. Both analyses were performed at least until 1 h after supplementation of Spm and drugs. Bar in **(A)** 100 μm; bars in **(B–D)** 20 μm.

The involvement of the microtubular cytoskeleton was also investigated to understand if it takes part in the swelling process. In control pollen tubes, no effects due to oryzalin or taxol treatment were observed and growth of pollen tubes was comparable to untreated ones in terms of growth rate and morphology (data not shown). Neither taxol (Figures 6A,B) nor oryzalin (Figures 6C,D) affected the morphological changes induced by Spm. Both timing of changes and shape were comparable in the presence of inhibitors to those observed in Spm-treated pollen tubes (Figures 6A,B). A statistical analysis showing the null effect of taxol and oryzalin on both morphology and growth rate of pollen tubes after Spm treatment is illustrated in the Supplementary Figure 3.

### Organelles in shovel-shaped pollen tubes move differently

**Figure 7:**
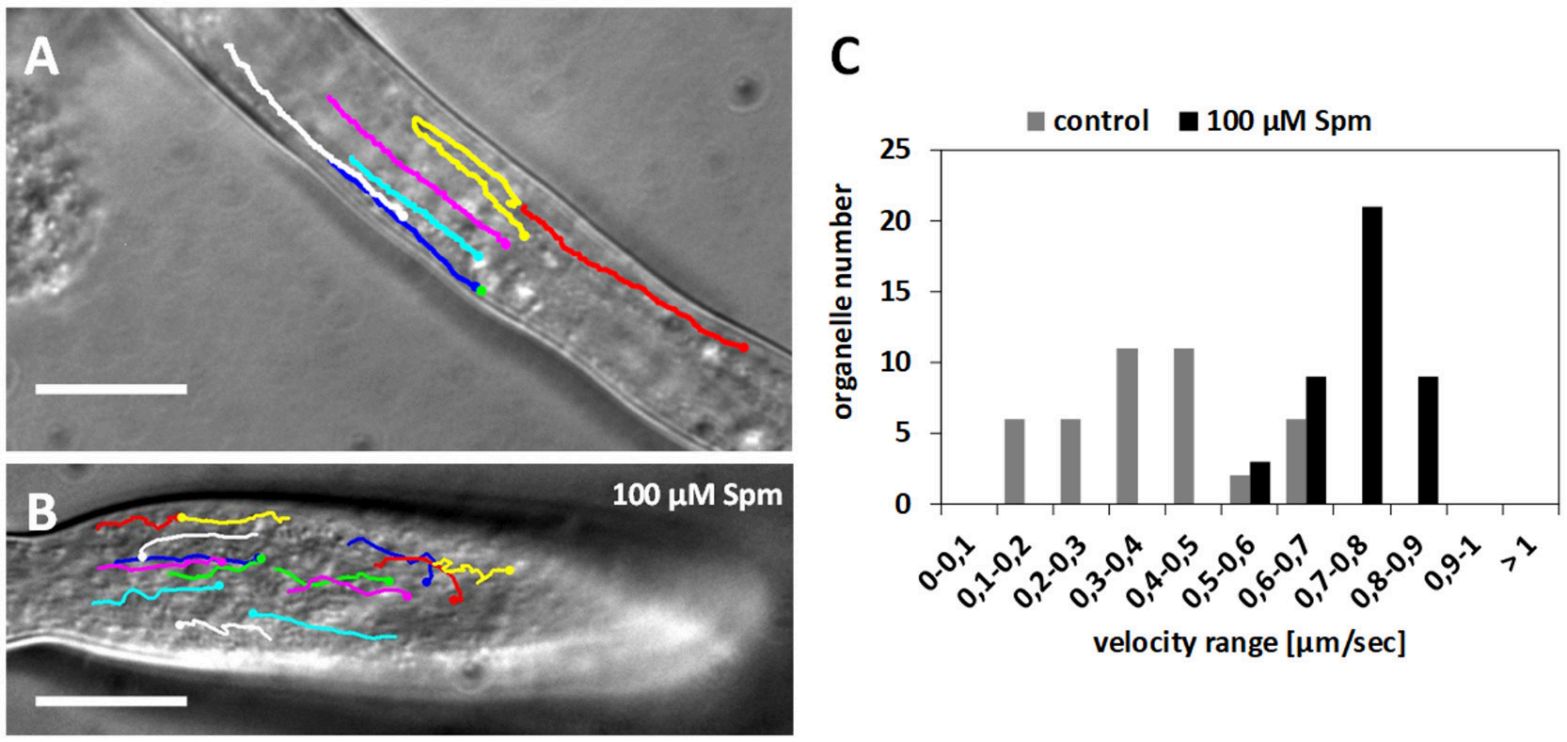
Velocity distribution of organelles in control and Spm-treated pear pollen tubes. **(A)** Control pollen tube with lines indicating the pathway of some monitored organelles. **(B)** Pathway of some organelles in a Spm-treated pollen tube. Bars: 10 μm. **(C)** Velocity distribution of representative organelles in control and Spm-treated pollen tubes. The Y-axis indicates the number of organelles while the X-axis indicates the velocity (in μm sec^−1^).

F-actin is the major structural factor supporting long-distance organelle transport in pollen tubes. Given that Spm stimulated F-actin to undergo major changes that involved actin depolymerization followed by the formation of thick actin filaments, alterations to organelle distribution and dynamics after Spm treatment were investigated. Observation of the shank region in normally-growing pollen tubes revealed rapid movement of organelles parallel to the longitudinal axis of the cell (Figure 7A). Larger organelles were rapidly transported toward the growing apex with speeds that could be clustered in a distinct range. Most of the organelles exhibited a speed of 0.3–0.5 μm s^−1^ but some specific organelles showed speeds up to 0.7 μm s^−1^ (histograms in Figure 7C). In the apical area, larger organelles were then transported back in a basipetal direction resuming the same speed as before. Small vesicles supposedly crossed this area and accumulated in the apex where they could not be distinguished clearly due to their small size (Figures 7A,B). After Spm supplementation, cytoplasmic streaming was not arrested (Figure 7B) and organelles moved significantly faster, with most exhibiting a speed of 0.8 μm s^−1^ (histograms in Figure 7C). Organelles followed the normal streaming direction seen in control pollen tubes; however, in most cases, they were marginalized in the cortical region, thus avoiding crossing the central expanded area (see Supplementary Movie 1). This sometimes produced a central stationary area in which many organelles moved slowly or not at all. Organelles moving quickly along actin cables could enter the stationary area, stopping once inside and then getting back on track (see Supplementary Movie 1).

### Proper accumulation of vesicles is required for Spm to expand the pollen tube apex

**Figure 8:**
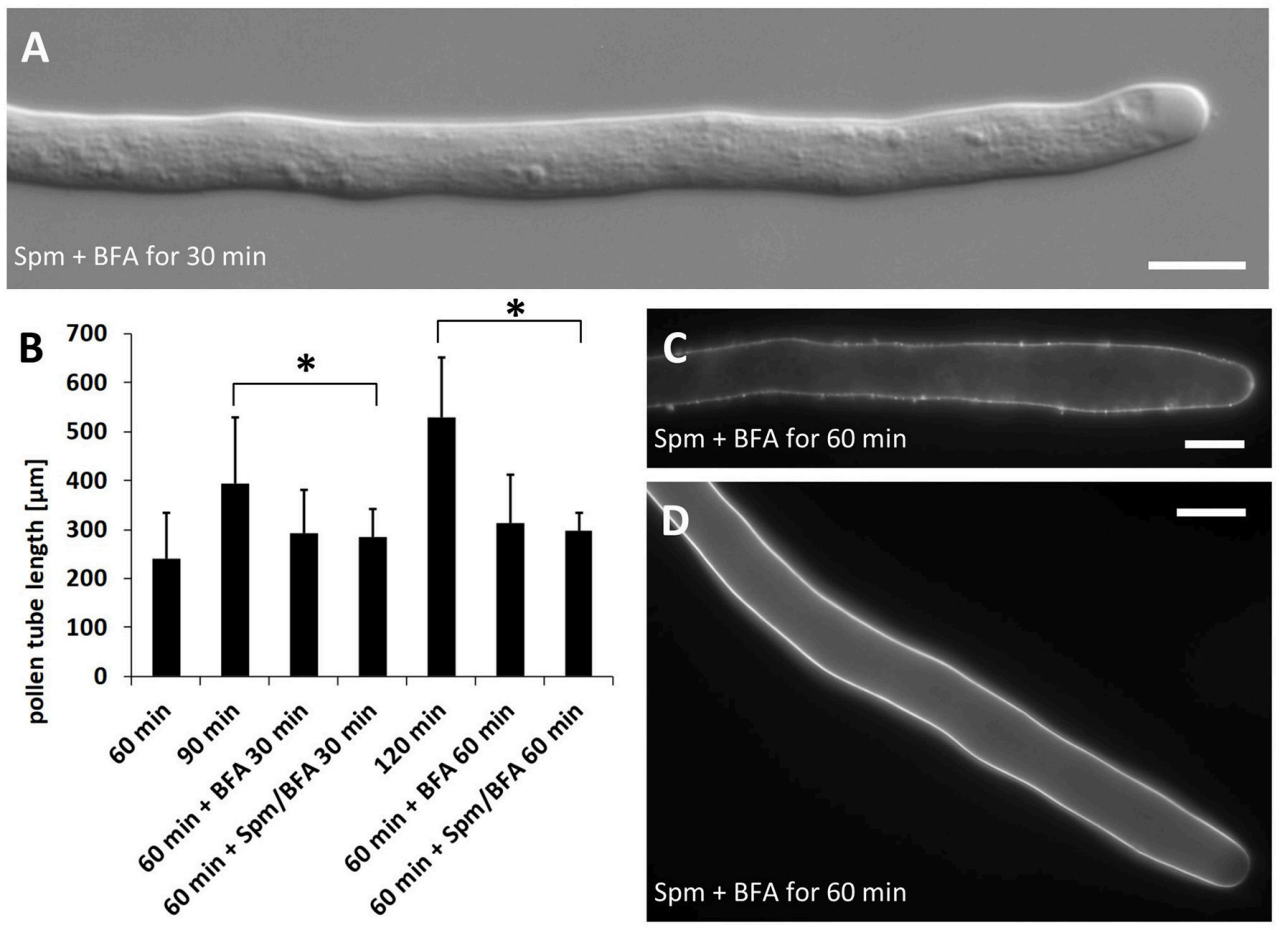
Effects of treatment with Brefeldin A (BFA) and Spm on pear pollen tube length and cell wall components. **(A)** A DIC view of 30 min-treated pollen tubes showing no defects in tube morphology. **(B)** Growth rates of control pollen tubes and of either BFA- or Spm+BFA-treated pollen tubes. The 60-min stage (first bar on the left) represents pollen tube growth before addition of chemicals. Pollen tubes were supplemented with BFA or Spm+BFA for an extra 30–60 min or they were grown under control conditions. The asterisk indicates that the measurements within square brackets are significantly different; in particular, data of treated pollen tubes are always significantly different compared to controls immediately to the left. Statistical analysis was performed using one-way ANOVA. **(C)** Labeling of pectins by PI. Pectins appear uniformly distributed. **(D)** Staining of callose by aniline blue. Callose is distributed as in controls and is absent in the very tip region. Bars: 10 μm.

Given that Spm drastically altered pollen tube morphology, the effects of the co-treatment with Spm and Brefeldin A (BFA) was investigated. The latter is known to affect the secretory and endocytotic pathways. As shown in Figure 8A, this combined treatment did not give rise to the typical balloon-shaped apex even after 30 min of treatment. Results were essentially the same until 60 min, suggesting an essential role of vesicle turnover in the process of apical swelling. As shown in Figure 8B, co-treatment with Spm and BFA prevented the expansion of the apical dome but also caused a strong reduction or even arrest of pollen tube growth. In the timeframe in which control pollen tubes had grown to about 100–150 μm, those treated with Spm+BFA grew much more slowly (not more than 20–30 μm after 1 h of treatment). As also indicated by the data in Figure 8B, the inhibitory effect on growth is most likely due to BFA; in addition, the ability of this molecule to inhibit pollen tube growth is known and described in the literature (Wang et al., 2005). Moreover, the co-treatment prevented deformation of the apex. Pollen tubes grown for 60 + 30 min with Spm+BFA were significantly different from those grown for 60 min or for 60 + 30 min in control conditions. On the contrary, pollen grown for 60 + 60 min with Spm+BFA showed no significant differences when compared to the sample treated for 30 min with Spm+BFA. This indicates that treatment with Spm+BFA affected growth during the first 30 min of incubation (Figure 8B). Again, the inhibitory effect on growth was due more to BFA because the presence of BFA only determined a significant reduction of growth.

### Spm alters the distribution of cell wall components

**Figure 9:**
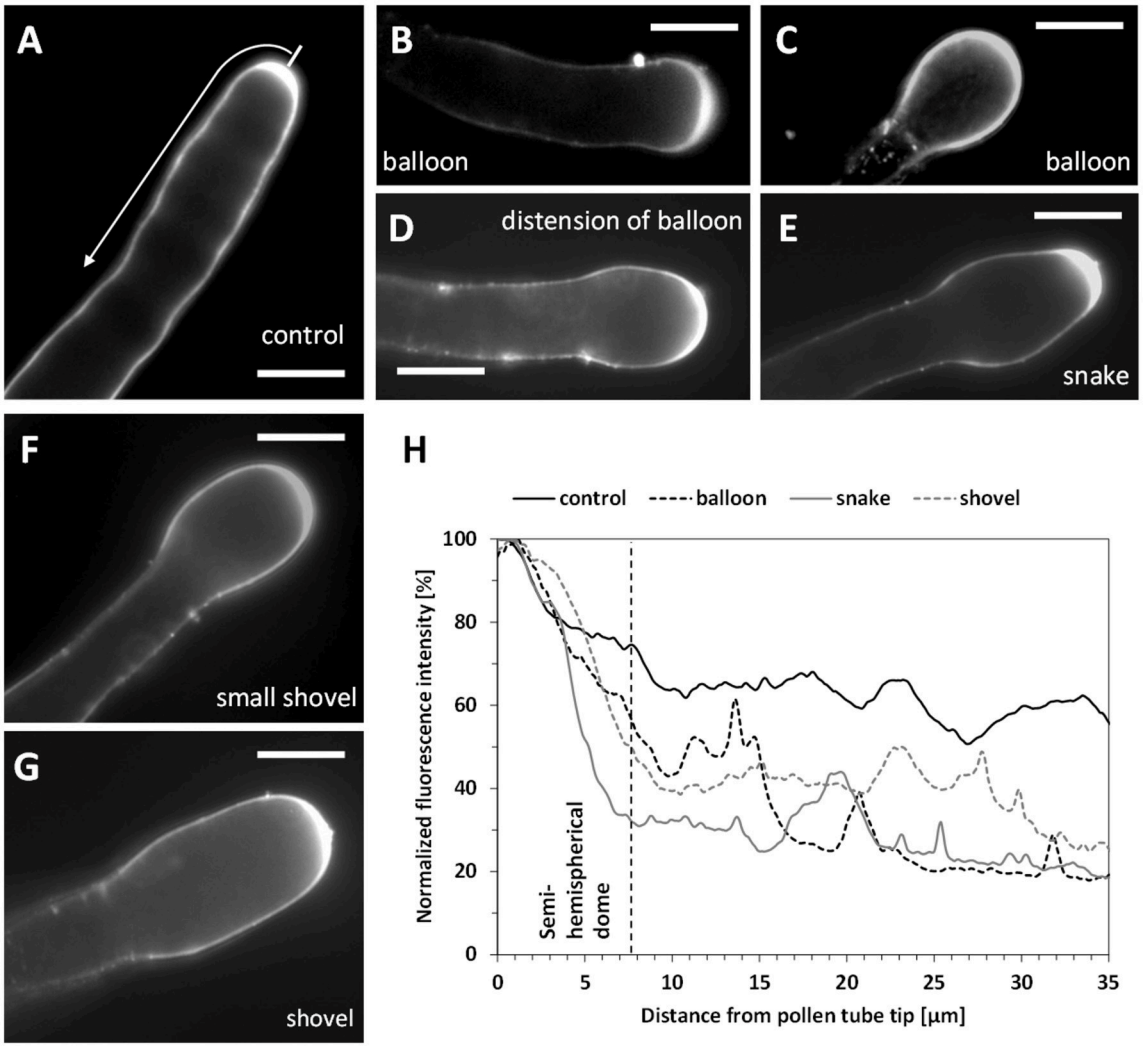
Pectin distribution in control and Spm-treated pear pollen tubes. **(A)** Distribution of PI-stained cell wall polysaccharides in control pollen tubes. Arrow indicates the surface analyzed for PI fluorescence intensity. **(B,C)** Balloon stage. **(D)** A pollen tube resuming growth and switching from the balloon to the snake stage. **(E)** A typical example of a snake-shaped pollen tube. **(F–G)** Initial stage of shovel formation and a mature shovel-shaped pollen tube, in which PI-labeled pectins accumulate again in the tip. Bars for all pictures: 10 μm. **(H)** Relative quantitation of PI fluorescence intensity in pollen tubes treated with Spm. The intensity profile is reported as relative fluorescence intensity starting from the tip. The analyzed half curvature of the pollen tube apex is called a “semi-hemispherical dome.” Graphs are calculated for the main steps of Spm treatment as indicated by the graph legend. Data are representative of three independent experiments.

Co-treatment with Spm and BFA also affected the deposition of cell wall components, as demonstrated by propidium iodide (PI), which vitally stains plant cell walls by binding to carboxyl residues on pectins. This allows the analysis of the link between cell wall deposition and cell growth. PI-stained pectins appeared uniformly distributed in the pollen tube border and no specific accumulation could be observed in the tip (Figure 8C; see Figure 9A below for control). Because pectins are secreted by secretory vesicles, this finding indicates that the accumulation and fusion of secretory vesicles might be altered. On the other hand, the deposition of callose in the cell wall was not modified (Figure 8D) because callose appeared as a uniform sheet surrounding the pollen tube except for the tip region.

The altered distribution of pectins by co-treatment with Spm and BFA may simply reflect the absence of tube growth. Therefore, the secretion of pectins in Spm-treated pollen tubes was investigated to check if the PA affected the deposition pattern of newly synthetized cell wall components. We measured the fluorescence intensity of PI from the tip down along the pollen tube perimeter (as shown in Figure 9A). Therefore, the values given below refer to the half of the pollen tube surface. In untreated pollen tubes, PI fluorescence was localized along the pollen tube walls mostly in a 7.2 ± 0.9 μm wide apical zone (Figure 9A; *n* = 40). After Spm application, at the balloon stage, newly deposited methoxylated pectins were no longer depleted from the subapical region and could be observed along a wider circumference (18.5 ± 1.6 μm; *n* = 35; Figure 9B). In some cases, staining with PI extended to the periphery of the entire tube portion involved in the formation of the swollen apex (Figure 9C). This suggests that the apex surface involved in the exocytosis of methoxylated pectins increased considerably in comparison with control pollen tubes. As pollen growth continued, distribution of PI fluorescence changed in Spm-treated pollen tubes; as soon as the balloon shape changed to the snake apex, the PI fluorescence was progressively refocused into a 7.4 ± 0.7 μm wide apical region (*n* = 35; Figures 9D,E), which represented the new growth site. When the newly-growing pollen tube reached the shovel shape, PI accumulated again, generating a new tip-focused secretion area of about 10.5 ± 0.8 μm (*n* = 40; Figures 9F,G). By measuring the relative fluorescence intensity in Spm-treated pollen tubes, the differential distribution of PI-labeled pectins concomitant with the typical morphological stages is shown in Figure 9H.

When pollen tubes were analyzed with JIM7 and JIM5 antibodies directed against high and low methyl-esterified pectins, respectively, the resulting data was consistent with that obtained by PI. In control samples (Supplementary Figure 4A), high methyl-esterified pectins were localized essentially in the apical region, where they are secreted. JIM5-labeled low methyl-esterified pectins (hereafter referred to as “acidic pectins”) showed a uniform pattern along the tube edge, including the apex (Supplementary Figure 4B). After treatment with Spm, at balloon stage the JIM7 signal expanded over the entire swollen surface of the tube apex, again suggesting that secretion involved a larger area (Supplementary Figure 4C). At a corresponding stage of treatment, the JIM5 signal was also found in the swollen apex but with decreased content in the tip (reasonably due to the higher secretion rate of esterified pectins; Supplementary Figure 4D). At the onset of shovel stage (Supplementary Figure 4E1), the JIM7 signal was relatively uniform in the bulged apex but, as soon as tube growth restarted, high methyl-esterified pectins again accumulated consistently in the apex (Supplementary Figure 4E2) with the signal level that rapidly decreased in older regions. Correspondingly, acidic pectins redistributed uniformly along the tube edge (Supplementary Figure 4F), including the apex, and this pattern was maintained for the entire duration of the shovel stage.

**Figure 10:**
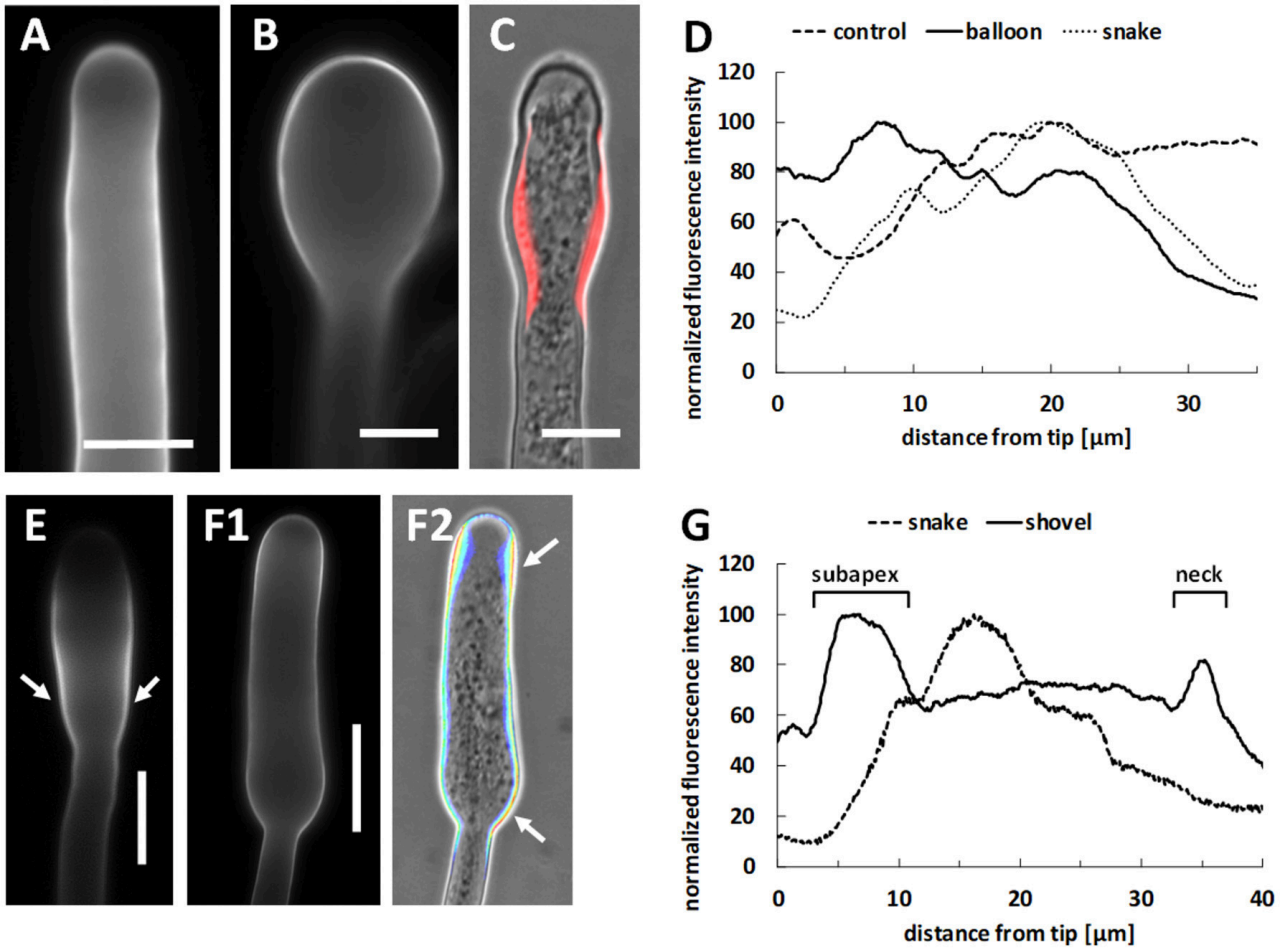
Distribution of cellulose in Spm-treated pollen tubes. **(A)** Pattern of cellulose in control pollen tubes. **(B)** Cellulose distribution at the onset of Spm treatment (balloon stage). **(C)** Distribution of cellulose at the snake stage; in this case, fluorescence of cellulose was thresholded to evince the sites of major accumulation and superimposed to the DIC view of the same pollen tube in order to emphasize the sites of cellulose accumulation. **(D)** Graph of relative fluorescence intensity at the balloon/snake transition (compared to control) starting from the tip. Data are representative of three independent experiments **(E)** Image of a pollen tube at the snake stage showing accumulation of cellulose in the neck and in enlarged regions (arrows). **(E,F1,F2)** Representative image of a pollen tube at the shovel stage. Cellulose accumulates all along the pollen tube but more prominently in the subapex and in the neck regions (arrows) as shown by pseudocolored fluorescence signal merged with the DIC view. Bars in **(A–C)** 10 μm. Bar in **(E,F1,F2)** 20 μm. **(G)** Graph reporting the relative fluorescence intensity in pollen tubes at the shovel stage starting from the tip. The so-called “enlargement zone” represents the point where the diameter of that specific pollen tube increases. Data are representative of three independent experiments

In control pollen tubes, cellulose appeared to be distributed according to a standard pattern characterized by a relatively uniform distribution along the pollen tube cell wall (Figure 10A). As soon as the pollen tube tip enlarged after Spm treatment, cellulose appeared as more or less uniformly deposited in the swollen apex (Figure 10B) and no specific accumulation of cellulose was observed. The cellulose pattern changed distinctly when pollen tubes shifted from the balloon to the snake stage. In this case, a prominent accumulation of cellulose was observed in the so-called neck region of swollen tubes (Figure 10C). Relative quantization of the fluorescence signal clearly showed that cellulose is quite uniformly distributed in control pollen tubes while it apparently increases in the swollen tip at the balloon stage; during the transition toward the snake stage, accumulation of cellulose was mainly detected ca. 20 μm from the tip (Figure 10D). When enlarged pollen tubes resumed growth (at the transition between snake and shovel stages), cellulose deposition was observed along the border of the shovel-shaped tube; deposition of cellulose was not uniform because a marked accumulation was still found in both the neck region and the swollen portion (arrows in Figure 10E). Later, at the shovel stage (Figure 10F1), cellulose was detected in the enlarged pollen tubes with a more consistent accumulation in the subapex and neck regions, as shown by pseudo-colored imaging of cellulose fluorescence merged with a DIC-imaged pollen tube (arrows in Figure 10F2). This result was confirmed by relative measurement of fluorescence intensity along the cell border (graph in Figure 10G) showing that, at the end of the snake stage, cellulose still peaked at 15–20 μm from the tip, while the shovel stage was characterized by two distinct accumulation areas, one in the subapex, the other in the neck region.

### Callose distribution is comparable to the cellulose pattern

**Figure 11:**
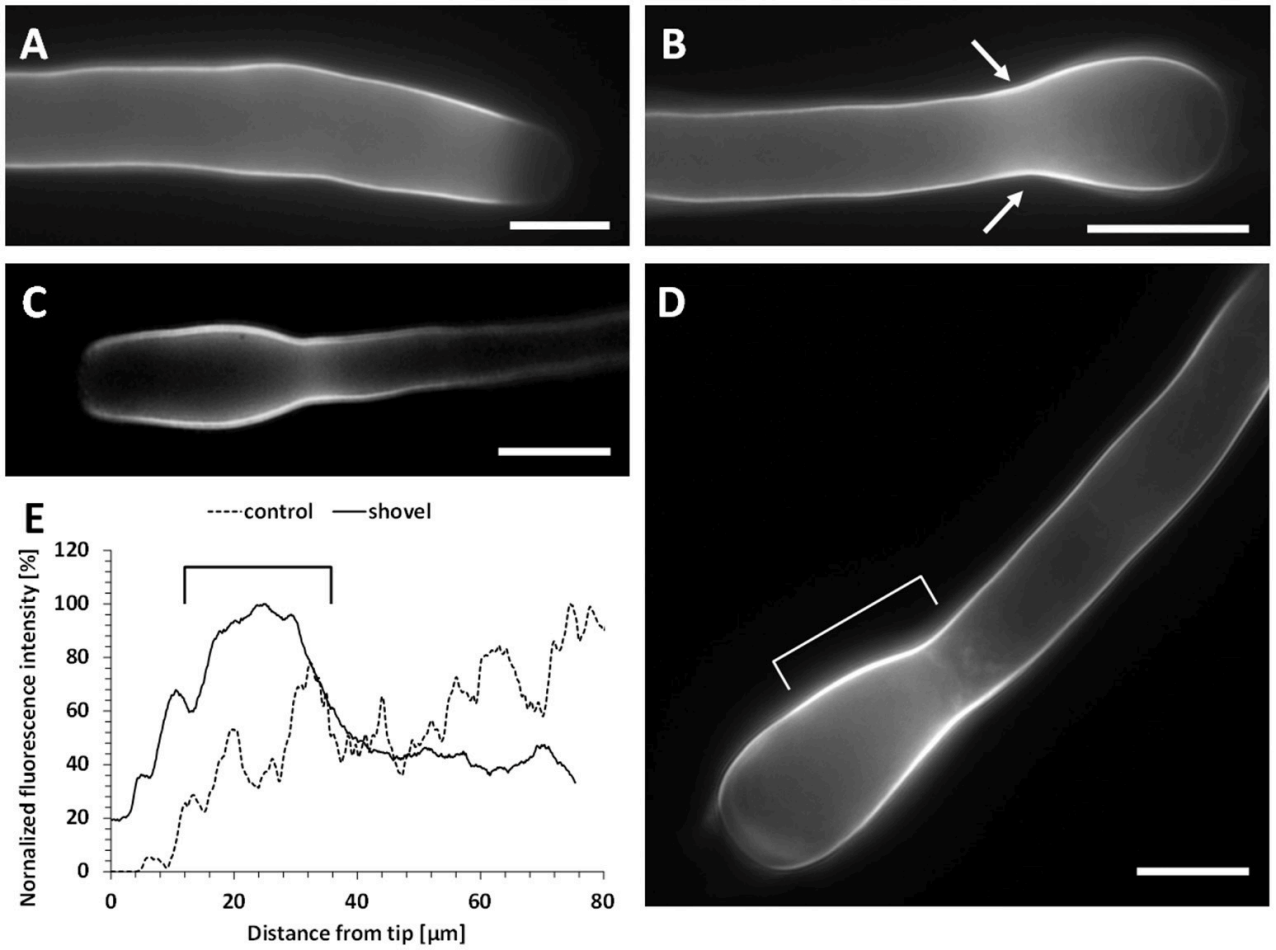
Distribution of callose in Spm-treated pear pollen tubes. **(A)** A control pollen tube showing absence of callose in the tip. **(B)** In balloon-shaped pollen tubes, callose accumulates in the neck region (arrows). **(C)** In snake-shaped pollen tubes, callose still accumulates in the neck region and it is still absent from the tip. **(D)** In shovel-shaped pollen tubes, callose showed a consistent accumulation around 20–30 μm, as also shown by the relative fluorescence intensity **(E)**. The square bracket in **(D)** corresponds to the region indicated by the square bracket in the graph of **(E)**. Bars: 10 μm. Data are representative of three independent experiments.

While in control pollen tubes callose was uniformly distributed except in the very tip region (Figure 11A), it accumulated in the neck of Spm-treated pollen tubes at the onset of treatment (Figure 11B, arrows). Accumulation of callose in the neck region was more prominent at the snake stage (Figure 11C); callose levels remained constantly higher in the neck during the transition from the snake to the shovel stage (Figure 11D, square bracket). This observation was confirmed by measuring the relative fluorescence intensity in pollen tubes at both control and shovel stages (graph in Figure 11E). While control pollen tubes exhibited no callose deposition in the tip and a progressive accumulation starting from 20 to 30 μm, shovel-shaped pollen tubes were characterized by a consistent accumulation of callose in the neck region, which corresponds to ca. 30 μm from the tip (Figures 11D,E).

### Changes to the callose pattern are accompanied by changes in callose synthase distribution

**Figure 12:**
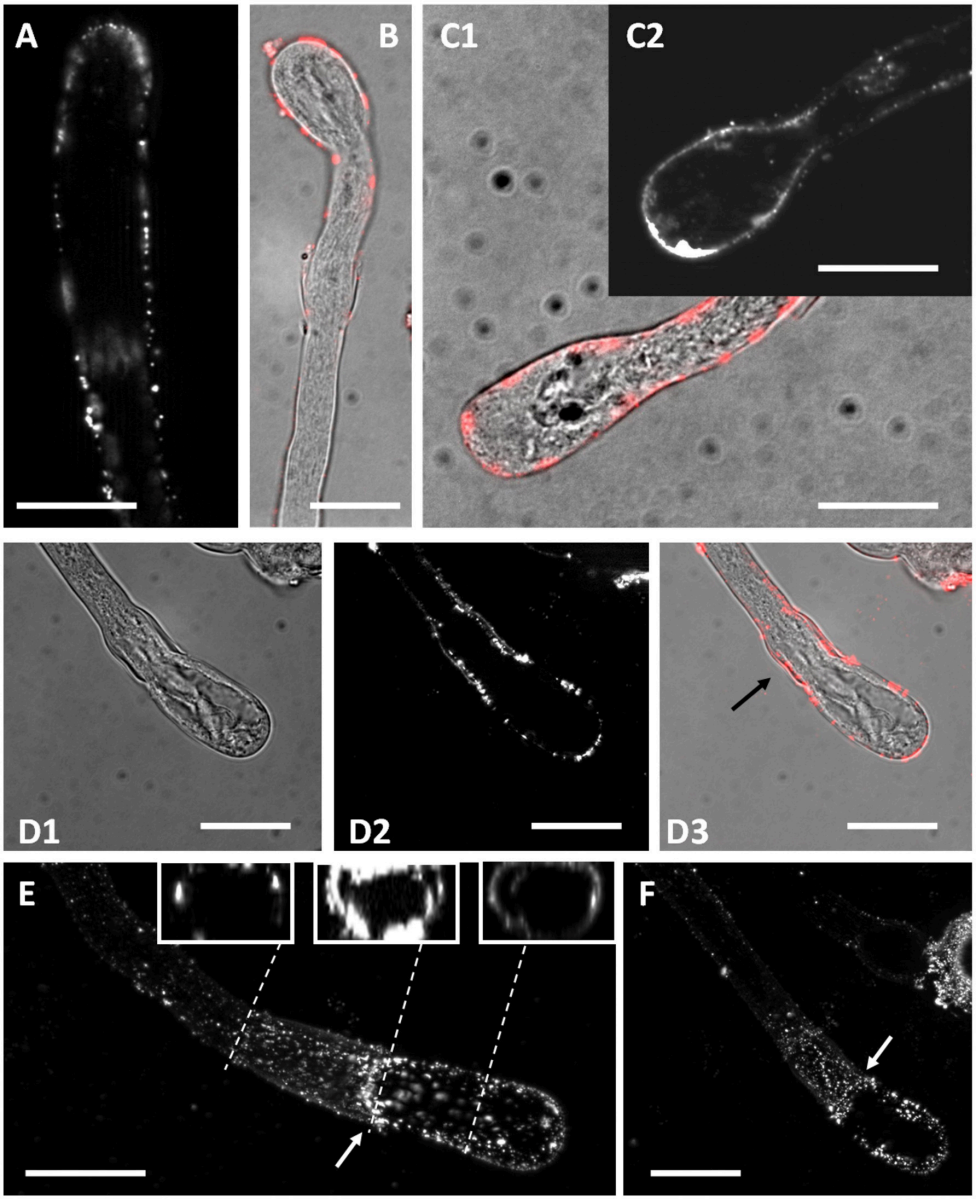
Distribution of callose synthase in control and Spm-treated pear pollen tubes. **(A)** Callose synthase is present as dots or patches along the entire border of control pollen tubes. **(B)** At the balloon-like step, callose synthase accumulates in the spherical domain. This image is a merge of phase contrast and immunofluorescence (red) pictures of the same pollen tube. **(C1,C2)** Accumulation of callose synthase in the apical domain is more evident at the transition between the balloon-like and the shovel-like step. The image in **(C1)** is a merge of immunolocalized callose synthase (red) with phase contrast view of the same pollen tube. The image in **(C2)** is an immunofluorescence view of another pollen tube. **(D1–D3)** Accumulation of callose synthase in the neck (arrow) becomes evident when pollen tubes develop into the snake shape and start assuming the shovel shape. **(E–F)** Two additional views of Spm-treated pollen tubes showing a consistent accumulation of callose synthase in the neck region (arrows), with an annulus-like configuration. Bars = 10 μm. The insets in Figure 11E show three reconstructions from a Z-series stack demonstrating that the annulus does not have uniform fluorescence intensity. Data are representative of three independent experiments.

As the distribution of callose was altered by Spm, we checked, by immunofluorescence microscopy, if this might depend on the irregular localization of callose synthase. As shown in Figure 12A, in control pollen tubes the enzyme was detected along the entire periphery of pollen tubes, most likely in the plasma membrane. Distribution of callose synthase was not uniform, but can be described as organized in patches, more abundant in the apical zone (the hypothetical insertion site). After Spm treatment, at the balloon stage, callose synthase accumulated in the plasma membrane of the swollen apex (Figure 12B) again with a patchy pattern. Labeling was not limited to the swollen apex but also extended to distal segments (Figures 12C1,C2). When pollen tubes resumed growth (snake stage), callose synthase started to accumulate consistently in the neck region (Figures 12D1–D3) thus mirroring the neck-localized deposition of callose.

In some cases, deposition of callose synthase took the form of annulus-like structures surrounding the neck region (arrows in Figures 12E,F). Three reconstruction images from a Z-stack showing the pollen tube regions before the annulus, at the annulus level and after the annulus are shown in the insets of Figure 12E. A significant amount of callose synthase accumulation is evident in the central inset (i.e., annulus). The annulus was not uniform but was characterized by relatively more or less intense areas. The so-called “annulus” was never observed in control samples. In samples treated with spermine, presence of the annulus was not an absolute feature but it was still very frequent. By counting the analyzed pollen tubes, it turned out that ~60–65% of pollen tubes showed the structure defined as annulus. Currently, we do not know why this structure is not present in all treated pollen tubes but we are confident that it is neither an artifact nor an incidental structure. A video clip built with a series of distal-to-proximal Z stacks (with respect to the tube tip) illustrating the presence of the callose synthase annulus is available as Supplementary Movie 2.

## Discussion

**Figure 13:**
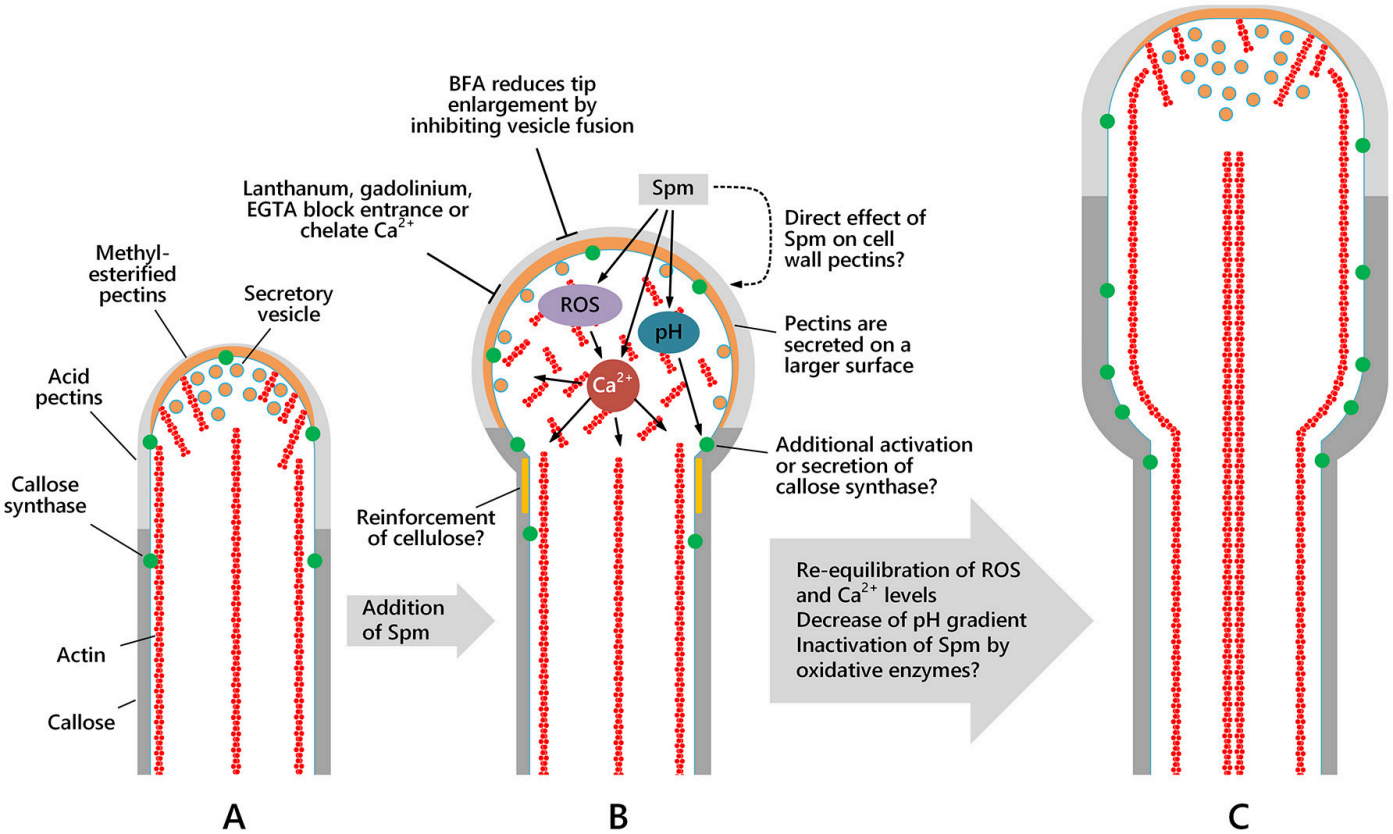
Schematic drawing showing the morphology of the apical region and the spatial distribution of actin filaments, secretory vesicles and cell wall components in pollen tubes and how Spm perturbs either their distribution or the morphology of the apical region. The pollen tube sub-apex is characterized by the actin fringe while in the shank region actin filaments form regular and longitudinal cables, which are essential for organelle and vesicle movement. Spm profoundly alters Ca^2+^, H^+^, and ROS concentrations and distribution, thereby affecting not only microfilament organization but also vesicle delivery. As a final result, assembly of the cell wall and shaping of the growing pollen tube tip is altered. **(A)** Pollen tube after 1 h of germination; **(B)** pollen tube after 1 h of germination in standard medium, then supplied with Spm for one additional hour; **(C)** pollen tube grown for 1 h in standard medium and then supplemented with Spm for two additional hours. As yet unknown mechanisms re-equilibrate ion distribution and concentration and allow the pollen tube to resume growth even though tube diameter remains larger and growth rate slower. Inactivation of Spm by oxidative enzymes cannot be excluded.

This work provides experimental evidence that Spm functions as a regulator of growth and shape of pollen tubes. The response to Spm is characterized by a series of modifications, both at the molecular and structural level, discussed below and summarized in Figure 13.

Spm, whose internalization in growing pollen tubes is very rapid (Aloisi et al., 2015), caused, in a few minutes, an irreversible enlargement of the pollen tube tip and a transient 10-fold decrease in growth rate. Then, after 15–20 min, the structural growth program was resumed and the enlarged diameter of the apex in the balloon stage was maintained in the growing snake and shovel stages (Figures 13B,C). Growth rate was, however, recovered only at half of its original value.

Present data confirms the relevant role of actin in the complex changes induced by Spm. Thus, longitudinal actin filaments were transformed into unorganized short ones at the apex of the balloon-shaped tube resembling those described when the actin cytoskeleton is altered by the actin stabilizer jasplakinolide (Cárdenas et al., 2005). The possibility that Spm exerted a direct effect on actin is supported by the fact that, in pollen, actin was found covalently linked to PAs by a Ca^2+^-dependent cytoplasmic transglutaminase (TGase; Del Duca et al., 1997, 2013). *In vivo*, this enzyme is present in the growing region of pear pollen tubes (Del Duca et al., 2013). Thus, in Spm-treated pollen, the activity of TGase may be enhanced by the concomitant increase of both Spm and [Ca^2+^]_cyt_, thereby affecting the proper polymerization of actin (Del Duca et al., 2009). In addition, it cannot be excluded that changes in [Ca^2+^]cyt may unbalance the regulation of ROP activity, thereby leading to extensive secretion and to the balloon stage (Yan et al., 2009; Qin and Yang, 2011).

In the balloon stage, the movement of organelles was subjected to an enhanced but disordered transport. If myosin activity was not altered, the new disorganized actin configuration could be responsible for the modified organelle transport. There is currently little information on the relationships between actin filament arrays and organelle speed. What is well known is that organelles move linearly toward the apex in the presence of longitudinal actin filaments and in a more disorderly manner at the level of the actin fringe (Lovy-Wheeler et al., 2007). The local organization of actin filaments is, therefore, responsible for the specific movement of organelles and vesicles (Kroeger et al., 2009).

Actin-based vesicle accumulation and fusion is strictly required for the appearance of the balloon shape in Spm-treated pear pollen tubes. In fact, the simultaneous addition of Spm and Brefeldin A, an inhibitor of protein transport from the endoplasmic reticulum to the Golgi apparatus (Parton et al., 2003), prevented swelling of the tube apex. Thus, although elongation rate was rapidly reduced in the presence of both molecules, the characteristic balloon shape was not observed, indicating the importance of secretory vesicle delivery to the apex for the remodeling of pollen tubes.

It is remarkable that microtubules are not apparently involved in the new growth and shape patterns exhibited by Spm-treated pollen tubes. This is consistent with the evidence that microtubules are not generally involved in the tip-growth of pollen tubes (Cai et al., 2015a). On the other hand, these data also suggest that Spm does not interfere with cellular processes mediated by microtubules. The precise role of microtubules in pollen tubes is not known, but they may be involved in the regulation of exo/endocytosis (Idilli et al., 2013) as well as in the deposition of cell wall polymers; therefore, Spm may not interfere with these specific processes.

Regrowth of pollen tubes after Spm treatment (i.e., the shift from the balloon to the snake stage) was characterized by the appearance of fine elongated actin filaments in the middle of the snake-shaped pollen apex, partial recovery of growth, and re-focusing of the secretion zone as shown by PI labeling. It is impossible to say if this implies the assembly of additional/supernumerary actin filaments or simply the organization of more radial actin arrays. Nevertheless, the new actin pattern supports an active streaming of organelles and vesicles and the recovery of growth, albeit with different features. It can be hypothesized that re-growth may be related either to the inactivation of Spm (e.g., by sequestration in the apoplast or binding to various cell components) or to its catabolism [e.g., by peroxisomal polyamine oxidases (PAOs) present in pollen; (Tavladoraki et al., 2016)].

Many of the above-described events may be linked or dependent on local changes in Ca^2+^ concentration and pH. In pollen treated with different PAs, we previously found that the Ca^2+^ gradient was rapidly dissipated and reconstituted (Aloisi et al., 2015). In the current work, the Ca^2+^-sensitive photoprotein aequorin fused to the cell-penetrating peptide TAT was used to measure the Spm-induced changes in [Ca^2+^]_cyt_. This sensitive and accurate technique has been previously successfully applied in suspension-cultured plant cells (Zonin et al., 2011) and in mycorrhizal fungi (Moscatiello et al., 2014; Salvioli et al., 2016). In control pollen tubes, the basal [Ca^2+^]_cyt_ was ca. 0.5 μM, which represents an average concentration in the tip-focused Ca^2+^ gradient along the pollen tube axis during growth. This [Ca^2+^]_cyt_ is an average value between a maximum [Ca^2+^]_cyt_ of 1–10 μM detected at the tip apex, depending on species and detection method (Holdaway-Clarke and Hepler, 2003), and a minimum [Ca^2+^]_cyt_ of 0.1–0.2 μM throughout the shank of the tube, measured with fluorescent Ca^2+^ probes (Steinhorst and Kudla, 2013; Hepler and Winship, 2015). Treatment with 100 μM Spm triggered a rapid and remarkable increase of [Ca^2+^]_cyt_, which was almost completely dissipated within 10 min. This is in agreement with the notion that Ca^2+^ is highly buffered in the cytoplasm (Michard et al., 2017). A two-fold increase in [Ca^2+^]_cyt_ was also induced by 1 mM spermidine in Arabidopsis pollen tubes as shown by the (FRET)-based Ca^2+^ indicator cameleon (Wu et al., 2010). Present results are thus in agreement with data indicating that PAs might affect intracellular Ca^2+^ levels likely as a consequence of the activation of plasma membrane Ca^2+^ channels (Pottosin and Shabala, 2014).

Even a transient change in cytosolic Ca^2+^ levels might affect different metabolisms or structures. It is known that Ca^2+^ regulates the dynamics of actin filaments through Ca^2+^-binding proteins (Zhang et al., 2010) as well as the PA binding to microfilaments that occur via TGase. An interconnection between Ca^2+^ and pH has to be taken into consideration, as pH changes might be caused by modulation of plasma membrane H^+^-ATPases; Spm, by causing a Ca^2+^ influx across the plasma membrane, can determine a Ca^2+^ uptake-synchronized imbalance between influx and efflux of H^+^ (Pottosin and Shabala, 2014; Pottosin et al., 2014). Moreover, a sudden but transient stimulation of the activities of several oxidative enzymes and an imbalance of reactive oxygen species (ROS) were observed in pear pollen tubes treated with different PAs (Aloisi et al., 2015). Optimal intracellular ROS levels, necessary for pollen tube growth, are controlled by different mechanisms and are strictly interconnected with Ca^2+^ concentration, with a positive feedback regulation (Potocky et al., 2007; Kaya et al., 2014; Lassig et al., 2014), which may enhance the responsiveness of pollen tubes to extracellular cues (Wudick and Feijo, 2014).

New information arising from the present work is that Spm also induces a profound redistribution of new cell wall components that are either secreted (like pectins) or deposited (like callose and cellulose). Pectins were secreted in a larger area leading to the balloon shape. Newly secreted methyl-esterified pectins refocused in narrow regions at the apex when pollen tubes transited from the balloon into the snake shape. Refocusing of pectin secretion at the extreme apex occurred simultaneously to the recovery of growth, supporting the hypothesis that this feature is necessary for growth, as already suggested by several authors (McKenna et al., 2009; Rojas et al., 2011; Bloch et al., 2016) but recently debated because of evidence pointing to the presence of an annulus (ring) of secretion 3–10 μm from the apex (Bove et al., 2008; Zonia and Munnik, 2008; Geitmann and Dumais, 2009; Stephan, 2017). Additional secretion of methyl-esterified pectins likely enhanced loosening of the tube cell wall leading to apical swelling under the internal turgor pressure. In fact, assuming that the turgor pressure does not change (Winship et al., 2011), the formation of swollen pollen tubes might depend on changes to the cell wall architecture. In fact, apical growth does not rely so much on turgor pressure as does elongation growth in other plant cells (Cosgrove, 2014; Ali and Traas, 2016; Michard et al., 2017).

Ca^2+^ plays an essential role in pectin net formation and stabilization. As PAs can directly bind to pectic substances by ionic linkages, they may compete with Ca^2+^ for the formation of intermolecular bridges, thereby affecting the net mesh architecture, its charge and the packing of polygalacturonic chains (D'orazi and Bagni, 1987; Berta et al., 1997; Lenucci et al., 2005). As soon as a new growing tip started to form, rearrangement of actin filaments may have refocused vesicle secretion. Two glucose-based polysaccharides, cellulose, and callose have been reported to regulate pollen tube shape by counteracting the internal turgor pressure (Aouar et al., 2010; Abercrombie et al., 2011). Here we show that both cellulose and callose levels in Spm-treated pollen tubes increased in the neck region and, subsequently, in the enlarged pollen tube. This finding could be interpreted as a response to the swelling process caused by the extension of the pectin deposition area, which weakened the cell wall structure. Therefore, increased cellulose accumulation might have counteracted the altered pectin secretion.

The differential deposition of callose in Spm-treated pollen tubes was mirrored by changes in the distribution pattern of callose synthase. Present findings suggest that the enlargement of the tube apex might induce the specific accumulation and activation of callose synthase in the neck region, whereas it possibly remains inactive in the apex as no callose was found there. Little is known about callose synthase activation and callose deposition. The enzyme is likely transported via Golgi-derived secretory vesicles along actin filaments (Brownfield et al., 2008; Cai et al., 2011), while its activation requires a lipid domain and, possibly, proteolytic cleavage (Brownfield et al., 2008). Evidence linking the activation of callose synthase to cell wall deformation in pollen tubes is lacking. Some information is available on the regulation of callose plug formation. Microtubules are reported to control the number of callose plugs in tobacco pollen tubes (Laitiainen et al., 2002) and are also possibly involved in the proper distribution of callose synthase in distal regions and around nascent callose plugs (Cai et al., 2011). In addition, formation of callose plugs is likely under the control of H^+^-ATPase activity and consequently of H^+^ flux into/out of pollen tubes (Certal et al., 2008).

A direct involvement of PAs in the reorganization of the cell wall is highly probable, as it is known that PAs are linked to cell wall molecules, *e.g*., hemicelluloses, lignin, and pectins (Aloisi et al., 2016). Moreover, in Rosaceae pollen, PAs are conjugated to cell wall proteins via an extracellular TGase forming protein polymers (Della Mea et al., 2007). Finally, PAs are known to be metabolized in the cell wall, notably by PAOs involved in the cross-linking of extensin and polysaccharide-bound phenols and in ROS production (Yoda et al., 2006; Angelini et al., 2010), consequently in wall loosening or stiffening (Swanson and Gilroy, 2010; Speranza et al., 2012).

Some papers report that Spm is not essential for survival of Arabidopsis plants at least under normal growth conditions (Imai et al., 2004). On the other hand, Spm might play a role in stress responses probably through the modulation of cation channel activities and as a source of hydrogen peroxide during pathogen infection. Conversely, thermospermine, an isomer of spermine, is likely involved in stem elongation, in repressing xylem differentiation and lateral root formation by modulating different genes expression among which those related to auxin signaling (Tong et al., 2014).

The interaction of polyamines with functionally diverse ion channels and receptors showed that the efficacy of PAs in modulating/blocking channel activities decreases according to the order spermine > spermidine > putrescine (Takahashi and Kakehi, 2010).

As experimental support for this hypothesis, the *spms* mutant appears to be more sensitive to drought and salt stresses than the wild-type and this phenotype might be related to the fact that inward potassium currents across the plasma membrane of guard-cells are blocked by intracellular polyamines (Liu et al., 2000). Blocking of ion channels by polyamines in plants has also been reported for vacuolar cation channels in barley and red beet and for non-selective cation channels in pea mesophyll cells (Dobrovinskaya et al., 1999; Shabala et al., 2007).

Since ion channel activities play a critical role during pollen tube elongation, the effect caused by Spm as reported in this manuscript can be reasonably attributed to this feature. Taken together our results highlight the possible involvement of Spm in the regulation of pollen tube growth and shaping via changes in cytosolic Ca^2+^ levels. If Spm has a similar effect *in planta* remains unknown, but if it had, this could be relevant for fertilization because a huge amount of PAs is released from the ovary after pollination.

## Author contributions

IA, SDD, and GC: Conceived the original screening and research plans; IA, CF, and LN: Performed most of the experiments; CF: Provided technical assistance; IA, SDD, and GC: Designed the experiments and analyzed the data; GC and DS-F: Wrote the article with contributions from all the authors.

### Conflict of interest statement

The authors declare that the research was conducted in the absence of any commercial or financial relationships that could be construed as a potential conflict of interest.

## Supplementary material

Supplementary Movie 1 Movement of organelles in the enlarged region of a pollen tube treated with 100 μM Spm. The circled area indicates a zone where organelles are standing for longer or shorter periods. Bar: 10 μm.

Supplementary Movie 2 A video clip created by the addition of various Z stacks, from the most distal (start of movie) toward the most proximal part of pollen tubes (end of movie), obtained by optical sectioning of pollen tubes labeled with anti-CalS. The video clip shows the presence of a callose synthase annulus. This structure can be noted about halfway through the video clip as a progressive increase of fluorescence in the cell cortex.

Supplementary Table 1 The perturbation of Ca^2+^ dynamics by externally applied La^3+^, Gd^3+^, or EGTA inhibits pollen tube elongation. Values marked with “a” are significantly different from controls based on Tukey's multiple comparison test at *P* < 0.05.

Supplementary Figure 1 Physiological and progressive decrease of pollen viability during *in vitro* germination **(A)** and concomitant increase in the basal [Ca^2+^]_cyt_ **(B)**.

Supplementary Figure 2 **(A)** Apical region of control pollen tube **(a)** or pollen treated with 100 μM La^3+^ **(b)**, 100 μM Gd^3+^ **(c)**, or 1 mM EGTA **(d)** showing no changes in external morphology. Bars: 10 μm. **(B)** Pretreatment with La^3+^, Gd^3+^, or EGTA counteracts, in a dose-dependent manner, the extension of the shovel-shaped apical region. **(a)** Pollen treated with 100 μM Spm only. **(b–d)** Pollen pretreated with 10, 25, and 50 μM La^3+^ 20 min prior to Spm supplementation. **(e–g)** Pollen pretreated with 10, 25, and 50 μM Gd^3^ 20 min prior to Spm supplementation. **(h,i,l)** pollen pretreated with 200, 400, and 1,000 μM EGTA 20 min prior to Spm supplementation. Bars: 30 μm.

Supplementary Figure 3 Morphological and growth data of pollen tubes treated with spermine and microtubule inhibitors (taxol and oryzalin). **(A)** Diameter of control pollen tubes (i.e., without microtubule inhibitors) and of pollen tubes treated with taxol/oryzalin as measured before and after addition of spermine (Spm). **(B)** Growth rate of control pollen tubes (without microtubule inhibitors) and of pollen tubes treated with taxol/oryzalin as measured before and after addition of Spm. Asterisks indicate statistically comparable differences before and after addition of Spm.

Supplementary Figure 4 (Left column) Labeling of pear pollen tubes with JIM7 antibody against high methyl-esterified pectins; (Right column) Labeling of pear pollen tubes with JIM5 antibody against low methyl-esterified (acidic) pectins. **(A,B)** control pollen tubes; **(C,D)** Spm-treated pollen tube at the balloon stage; **(E1,E2,F)** Spm-treated pollen tube at the shovel stage. Bars: 10 μm.
