# Supplementary material for: Spermine Regulates Pollen Tube Growth by Modulating Ca2+-Dependent Actin Organization and Cell Wall Structure
Source: Front Plant Sci. 2017 Sep 29;8:1701. doi: 10.3389/fpls.2017.01701 (PMC5627395; doi:10.3389/fpls.2017.01701)
Supplement: Supplementary Table 1 — The perturbation of Ca2+ dynamics by externally applied La3+, Gd3+, or EGTA inhibits pollen tube elongation. Values marked with “a” are significantly different from controls based on Tukey's multiple comparison test at P < 0.05. [file Table1.DOCX]

|  | **Concentration (μM)** | **Tube length (μm)** |  | **N° of counted pollens** |
| --- | --- | --- | --- | --- |
|  |  |  |  |  |
| **Cntr** | 0 | 413,9 |  | 106 |
| **LaCl3** | 1 | 395,5 |  | 115 |
| **LaCl3** | 10 | 337,0 | *a* | 120 |
| **LaCl3** | 50 | 259,0 | *a* | 108 |
| **LaCl3** | 100 | 269,7 | *a* | 111 |
| **GdCl3** | 1 | 385,8 |  | 106 |
| **GdCl3** | 10 | 314,8 | *a* | 107 |
| **GdCl3** | 50 | 236,3 | *a* | 108 |
| **GdCl3** | 100 | 233,1 | *a* | 100 |
| **EGTA** | 400 | 297,6 | *a* | 118 |
| **EGTA** | 1000 | 239,0 | *a* | 110 |
| **EGTA** | 5000 | 242,2 | *a* | 113 |
