# Supplementary figures and images for: Spermine Regulates Pollen Tube Growth by Modulating Ca2+-Dependent Actin Organization and Cell Wall Structure
Source: Front Plant Sci. 2017 Sep 29;8:1701. doi: 10.3389/fpls.2017.01701 (PMC5627395; doi:10.3389/fpls.2017.01701)

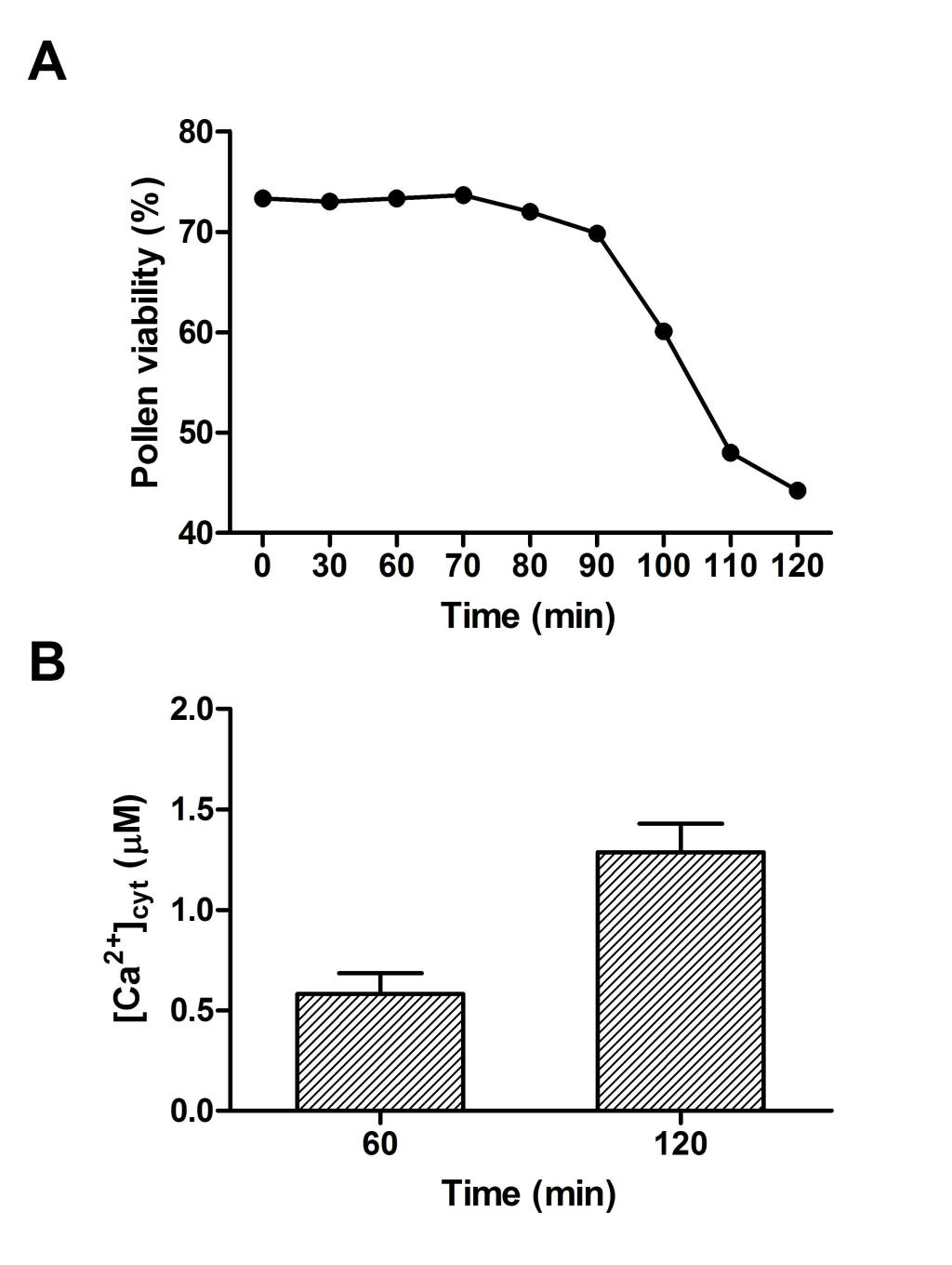

Supplement: Supplementary Figure 1 — Physiological and progressive decrease of pollen viability during in vitro germination (A) and concomitant increase in the basal [Ca2+]cyt (B). [file Image1.TIF]

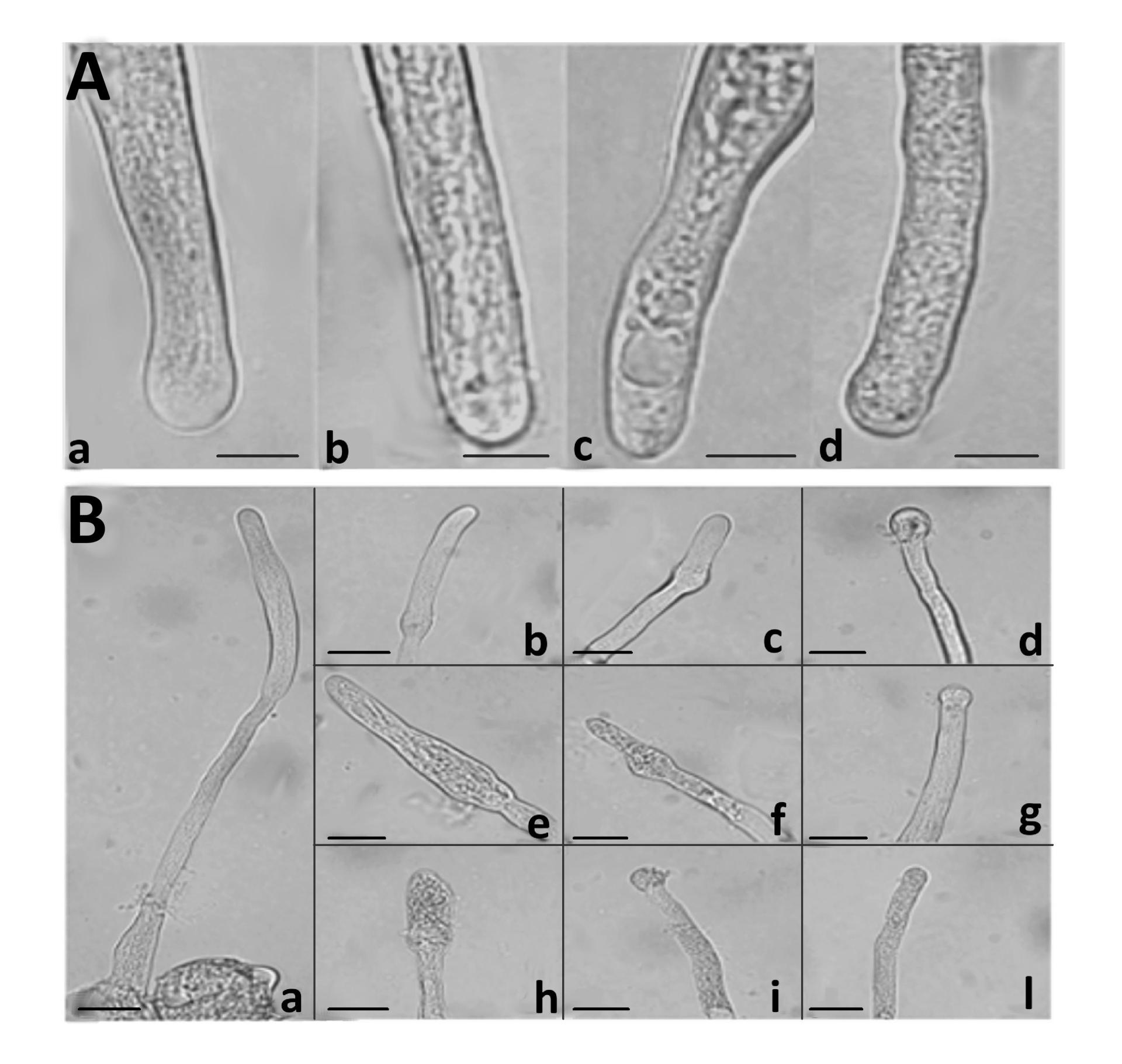

Supplement: Supplementary Figure 2 — (A) Apical region of control pollen tube (a) or pollen treated with 100 μM La3+ (b), 100 μM Gd3+ (c), or 1 mM EGTA (d) showing no changes in external morphology. Bars: 10 μm. (B) Pretreatment with La3+, Gd3+, or EGTA counteracts, in a dose-dependent manner, the extension of the shovel-shaped apical region. (a) Pollen treated with 100 μM Spm only. (b–d) Pollen pretreated with 10, 25, and 50 μM La3+ 20 min prior to Spm supplementation. (e–g) Pollen pretreated with 10, 25, and 50 μM Gd3 20 min prior to Spm supplementation. (h,i,l) pollen pretreated with 200, 400, and 1,000 μM EGTA 20 min prior to Spm supplementation. Bars: 30 μm. [file Image2.TIF]

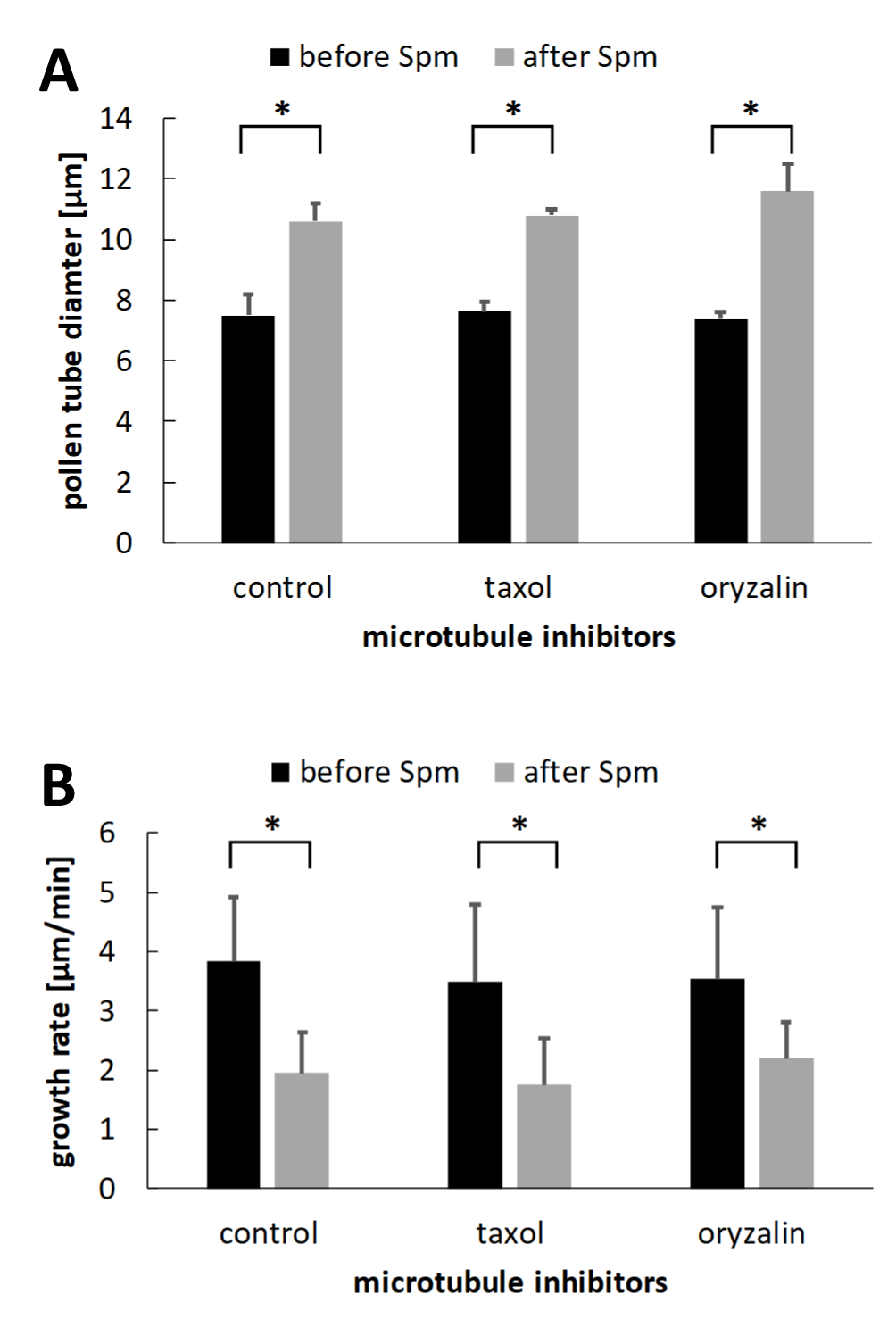

Supplement: Supplementary Figure 3 — Morphological and growth data of pollen tubes treated with spermine and microtubule inhibitors (taxol and oryzalin). (A) Diameter of control pollen tubes (i.e., without microtubule inhibitors) and of pollen tubes treated with taxol/oryzalin as measured before and after addition of spermine (Spm). (B) Growth rate of control pollen tubes (without microtubule inhibitors) and of pollen tubes treated with taxol/oryzalin as measured before and after addition of Spm. Asterisks indicate statistically comparable differences before and after addition of Spm. [file Image3.TIF]

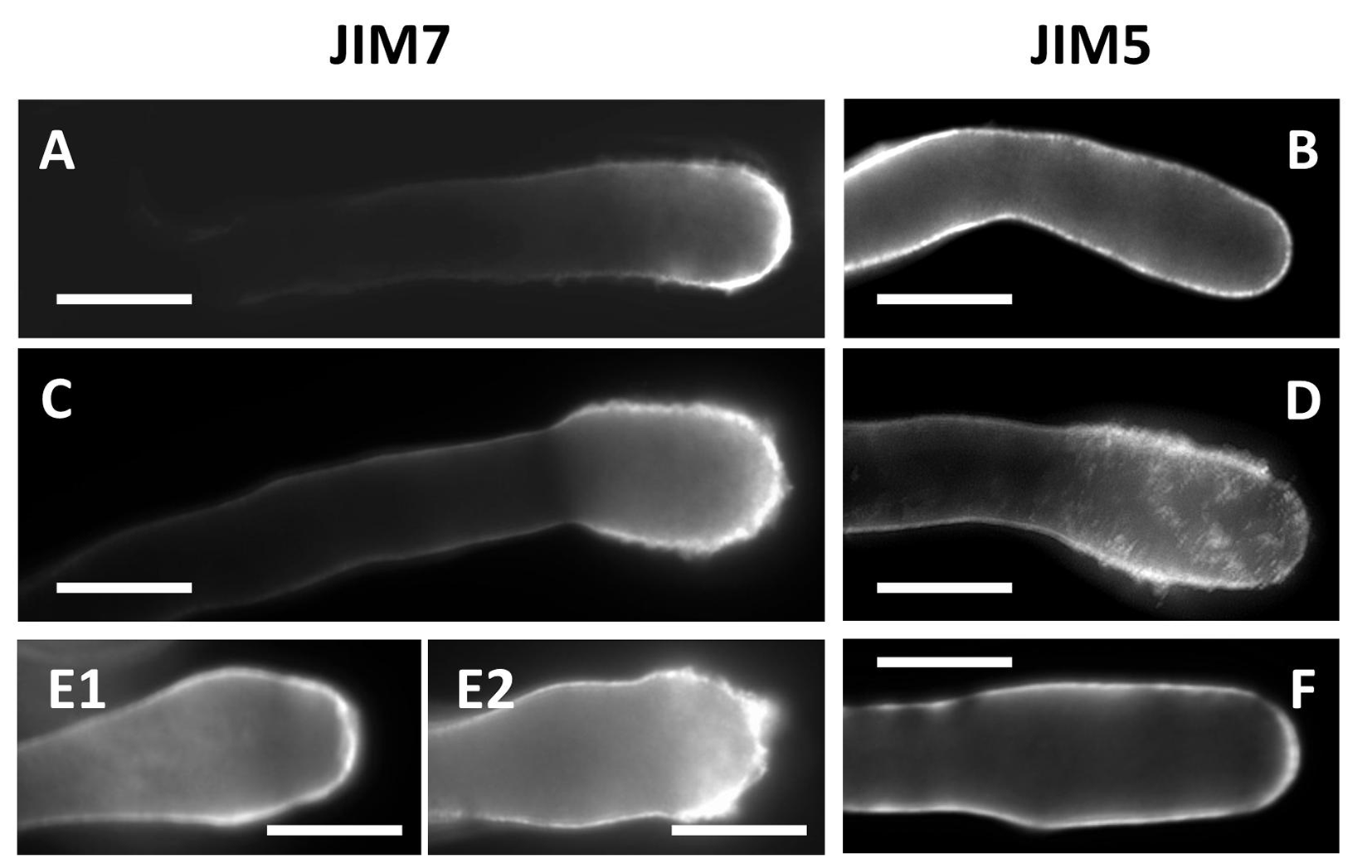

Supplement: Supplementary Figure 4 — (Left column) Labeling of pear pollen tubes with JIM7 antibody against high methyl-esterified pectins; (Right column) Labeling of pear pollen tubes with JIM5 antibody against low methyl-esterified (acidic) pectins. (A,B) control pollen tubes; (C,D) Spm-treated pollen tube at the balloon stage; (E1,E2,F) Spm-treated pollen tube at the shovel stage. Bars: 10 μm. [file Image4.TIF]
